# Supplementary material for: Reducing competition between msd and genomic DNA improves retron editing efficiency
Source: EMBO Rep. 2024 Nov 5;25(12):5316–30. doi: 10.1038/s44319-024-00311-6 (PMC11624263; doi:10.1038/s44319-024-00311-6)
Supplement: Supplementary file 1 — Appendix [file 44319_2024_311_MOESM1_ESM.pdf]

Appendix

Reducing competition between msd and genomic DNA improves retron editing efficiency

Ni et al.

Table of contents

Appendix Figure S1.
p15APSlacZ-anti sequence and map P. 3-4

Appendix Figure S2.
p15APSlacZ sequence and map P. 5-6

Appendix Figure S3.
pSClacZ sequence and map P. 7-8

Appendix Figure S4.
pUClacZ sequence and map P. 9-10

Appendix Figure S5.
p15AlacZ-lacZ sequence and map P. 11-12

Appendix Figure S6.
pBR-PkanX sequence and map P. 13-14

Appendix Figure S7.
p15A-PkanY sequence and map P. 15-16

Appendix Figure S8.
pBR-ØkanX sequence and map P. 17-18

Appendix Figure S9
p15A-ØkanY sequence and map P. 19-20

Appendix Figure S10.
pUClacZ1 sequence and map P. 21-22

Appendix Figure S11.
pUClacZ2 sequence and map P. 23-24

Appendix Figure S12.
pUClacZ3 sequence and map P. 25-26

Appendix Figure S13.
pUCung sequence and map P. 27-28

Appendix Figure S14.
pUCbetI sequence and map P. 29-30

Appendix Figure S1 | p15APSlacZ-anti sequence and map

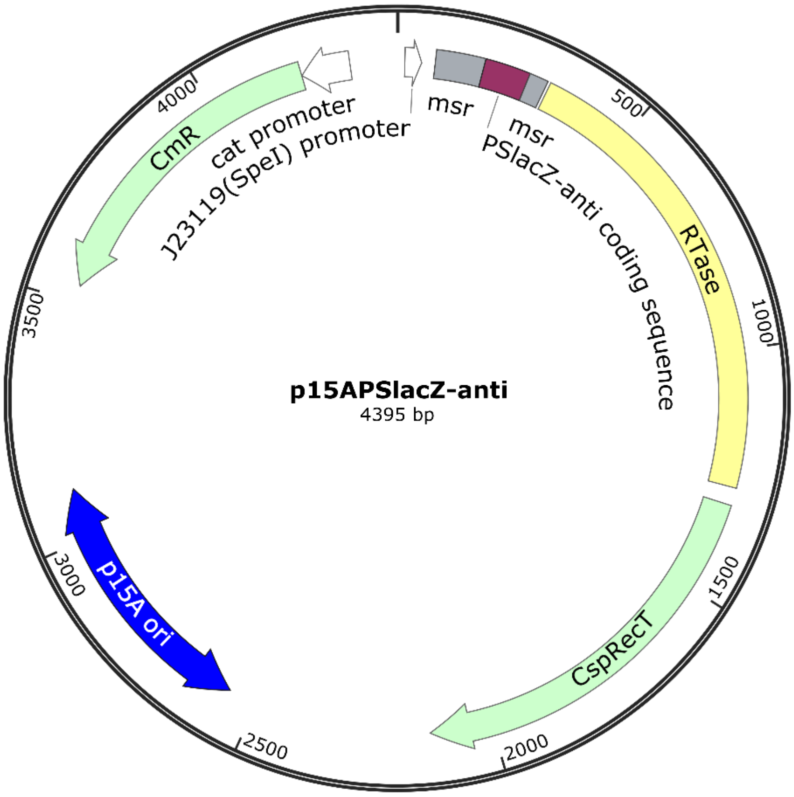

ctcgagatgctagcaattgacagctagctcagtcctaggtataatactagtatcagcaggacgcactgaccttaattaaatgcgcacccttagcgagaggtttatcattaaggtc
aacctctggatgttgtttcggcatcctgcattgaatctgagttactgtctgttttcctgaattcccatgattacggattcactggccgtcgttttacaacgtcgtgactgataaaaccc
tggcgttacccaacttaatcgccttgcagcacatccccaattcaggaaacccgtttttctgacgtaagggtgcgcaactttcatgaaatccgctgaatatttgaacacttttagat
tgagaaatctcggcctacctgtcatgaacaatttgcatgacatgtctaaggcgactcgcatatctgttgaaacacttcggttgttaatctatacagctgattttcgctataggatcta
cactgtagaaaagaaaggcccagagaagagaatgagaaccatttaccaaccttctcgagaacttaaagccttacaaggatgggttctacgtaacattttagataaactgtcgt
catctcctttttctattggatttgaaaagcaccaatctattttgaataatgctaccccgcatattggggcaaactttatactgaatattgatttggaggatttttttcccaagtttaactgct
aacaaagtttttggagtgttccattctcttggttataatcgactaatatcttcagttttgacaaaaatatgttgttataaaaatctgctaccacaaggtgctccatcatcacctaaattag
ctaatctaatatgttctaaacttgattatcgtattcagggttatgcaggtagtcggggcttgatatatacgagatatgccgatgacctcaccttatctgcacagtctatgaaaaaggt
tgttaaagcacgtgatttttttattttctataatcccaagtgaaggattggttattaactcaaaaaaaacttgtattagtgggcctcgtagtcagaggaaagttacaggtttagttatttc
acaagagaaagttgggataggtagagaaaaatataaagaaattagagcaaagatacatcatatattttgcggtaagtcttctgagatagaacacgttaggggatggttgtcattt
attttaagtgtggattcaaaaagccataggagattaataacttatattagcaaattagaaaaaaaatatggaaagaaccctttaaataaagcgaagacctaaggatccggttgat
attgattcagaggtataaaacgaatgaaccaaatcgtgaagttcactgacgactctggcctggcggttcaagttactccagacgatgttcgccgttatatctgtgagaacgcta
ctgaaaaagaggtgggcctctttctgcaactctgtcagactcaacgtctgaatccgtttgtgaaagacgcttacctggtgaaatacggcggtgctccagcttctatgattacttcc
tatcaagtttttaaccgtcgcgcgtgtcgtgatgctaactatgatggtatcaaatctggtgtggttgttctgcgtgacggtgatgttgtgcataaacgtggtgctgcgtgctacaaa
aaggcgggtgaggagctcatcggtggttgggcggaagttcgctttaaggatggccgcgagactgcgtatgctgaggtggcgctcgacgactattccaccggcaaatctaat
tgggcgaaaatgccgggtgttatgatcgaaaaatgcgcgaaggctgctgcttggcgcctcgcgttcccggacacttttcagggcatgtacgctgcggaggaaatggatcaa
gcgcaacagccagaacaggtgcgcgctcaggcggagcaaccagtggatctccagccaatccgcgaactcttcaagccatattgcgaacacttcggcatcactccggctg
agggtatgactgctgtttgtggtgcggtgggcgctgaaggcatgcactctatgaccgagcagcaagctcgccgtgctcgcgcttggatggaggaagaaatggctgcgcca
gctgtggaagcggagtatgaggttgttgacgagggcgaggtgttttaaacgcgtgctagaggcatcaaataaaacgaaaggctcagtcgaaagactgggcctttcgttttat
ctgttgtttgtcggtgaacgctctcctgagtaggacaaatccgccgccctagacctaggcgttcggctgcggcgagcggtatcagctcactcaaaggcggtaatacggttatc
cacagaatcaggggataacgcaggaaagaacatgtgagcaaaaggccagcaaaaggccaggaaccgtaaaaaggccgcgttgctggcgttagaatatgtgatacagga
tatattccgcttcctcgctcactgactcgctacgctcggtcgttcgactgcggcgagcggaaatggcttacgaacggggcggagatttcctggaagatgccaggaagatactt
aacagggaagtgagagggccgcggcaaagccgtttttccataggctccgcccccctgacaagcatcacgaaatctgacgctcaaatcagtggtggcgaaacccgacagg
actataaagataccaggcgtttccccctggcggctccctcgtgcgctctcctgttcctgcctttcggtttaccggtgtcattccgctgttatggccgcgtttgtctcattccacgcct
gacactcagttccgggtaggcagttcgctccaagctggactgtatgcacgaaccccccgttcagtccgaccgctgcgccttatccggtaactatcgtcttgagtccaacccg

gaaagacatgcaaaagcaccactggcagcagccactggtaattgatttagaggagttagtcttgaagtcatgcgccggttaaggctaaactgaaaggacaagttttggtgac
tgcgctcctccaagccagttacctcggttcaaagagttggtagctcagagaaccttcgaaaaaccgccctgcaaggcggttttttcgttttcagagcaagagattacgcgcag
accaaaacgatctcaagaagatcatcttattaatcagataaaatatttctagatttcagtgcaatttatctcttcaaatgtagcacctgaagtcagccccatacgatataagttgtaat
tctcatgttagtcatgccccgcgcccaccggaaggagctgactgggttgaaggctctcaagggcatcggtcgagatcccggtgcctaatgagtgagctaacttacattaattg
cgttgcgcgagctgaagcacacggtcacactgcttccggtagtcaataaaccggtaaaccagcaatagacataagcggctatttaacgaccctgccctgaaccgacgacc
gggtcgaatttgctttcgaatttctgccattcatccgcttattatcacttattcaggcgtagcaccaggcgtttaagggcaccaataactgccttaaaaaaattacgccccgccctg
ccactcatcgcagtactgttgtaattcattaagcattctgccgacatggaagccatcacagacggcatgatgaacctgaatcgccagcggcatcagcaccttgtcgccttgcgt
ataatatttgcccatagtgaaaacgggggcgaagaagttgtccatattggccacgtttaaatcaaaactggtgaaactcacccagggattggctgagacgaaaaacatattct
caataaaccctttagggaaataggccaggttttcaccgtaacacgccacatcttgcgaatatatgtgtagaaactgccggaaatcgtcgtggtattcactccagagcgatgaaa
acgtttcagtttgctcatggaaaacggtgtaacaagggtgaacactatcccatatcaccagctcaccgtctttcattgccatacggaactccggatgagcattcatcaggcggg
caagaatgtgaataaaggccggataaaacttgtgcttatttttctttacggtctttaaaaaggccgtaatatccagctgaacggtctggttataggtacattgagcaactgactga
aatgcctcaaaatgttctttacgatgccattgggatatatcaacggtggtatatccagtgatttttttctccattttagcttccttagctcctgaaaatctcgataactcaaaaaatacg
cccggtagtgatcttatttcattatggtgaaagttggaacctcttacgtgccgatcaacgtctcattttcgccagatatcgacgtctaagaaaccattattatcatgacattaacctat
aaaaataggcgtatcacgaggccctttcgtcttcac

Appendix Figure S2 | p15APSlacZ sequence and map

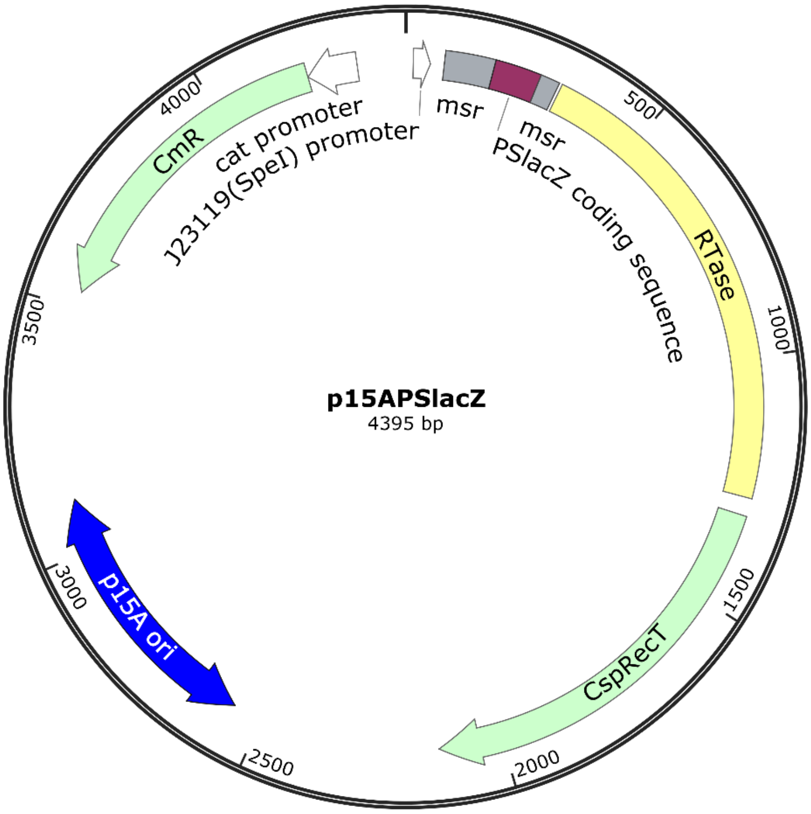

ctcgagatgctagcaattgacagctagctcagtcctaggtataatactagtatcagcaggacgcactgaccttaattaaatgcgcacccttagcgagaggtttatcattaaggtc
aacctctggatgttgtttcggcatcctgcattgaatctgagttactgtctgttttcctgaattcggggatgtgctgcaaggcgattaagttgggtaacgccagggttttatcagtcac
gacgttgtaaaacgacggccagtgaatccgtaatcatggaattcaggaaacccgtttttctgacgtaagggtgcgcaactttcatgaaatccgctgaatatttgaacacttttag
attgagaaatctcggcctacctgtcatgaacaatttgcatgacatgtctaaggcgactcgcatatctgttgaaacacttcggttgttaatctatacagctgattttcgctataggatc
tacactgtagaaaagaaaggcccagagaagagaatgagaaccatttaccaaccttctcgagaacttaaagccttacaaggatgggttctacgtaacattttagataaactgtc
gtcatctcctttttctattggatttgaaaagcaccaatctattttgaataatgctaccccgcatattggggcaaactttatactgaatattgatttggaggatttttttcccaagtttaactg
ctaacaaagtttttggagtgttccattctcttggttataatcgactaatatcttcagttttgacaaaaatatgttgttataaaaatctgctaccacaaggtgctccatcatcacctaaatt
agctaatctaatatgttctaaacttgattatcgtattcagggttatgcaggtagtcggggcttgatatatacgagatatgccgatgacctcaccttatctgcacagtctatgaaaaa
ggttgttaaagcacgtgatttttttattttctataatcccaagtgaaggattggttattaactcaaaaaaaacttgtattagtgggcctcgtagtcagaggaaagttacaggtttagtta
tttcacaagagaaagttgggataggtagagaaaaatataaagaaattagagcaaagatacatcatatattttgcggtaagtcttctgagatagaacacgttaggggatggttgtc
atttattttaagtgtggattcaaaaagccataggagattaataacttatattagcaaattagaaaaaaaaatatggaaagaacccttttaaataaagcgaagacctaaggatccggtt
gatattgattcagaggtataaaacgaatgaaccaaatcgtgaagttcactgacgactctggcctggcggttcaagttactccagacgatgttcgccgttatatctgtgagaacg
ctactgaaaaagaggtgggcctctttctgcaactctgtcagactcaacgtctgaatccgtttgtgaaagacgcttacctggtgaaatacggcggtgctccagcttctatgattact
tcctatcaagtttttaaccgtcgcgcgtgtcgtgatgctaactatgatggtatcaaatctggtgtggttgttctgcgtgacggtgatgttgtgcataaacgtggtgctgcgtgctac
aaaaaggcgggtgaggagctcatcggtggttgggcggaagttcgctttaaggatggccgcgagactgcgtatgctgaggtggcgctcgacgactattccaccggcaaatc
taattgggcgaaaatgccgggtgttatgatcgaaaaatgcgcgaaggctgctgcttggcgcctcgcgttcccggacacttttcagggcatgtacgctgcggaggaaatggat
caagcgcaacagccagaacaggtgcgcgctcaggcggagcaaccagtggatctccagccaatccgcgaactcttcaagccatattgcgaacacttcggcatcactccgg
ctgagggtatgactgctgtttgtggtgcggtgggcgctgaaggcatgcactctatgaccgagcagcaagctcgccgtgctcgcgcttggatggaggaagaaatggctgcg
ccagctgtggaagcggagtatgaggttgttgacgagggcgaggtgttttaaacgcgtgctagaggcatcaaataaaacgaaaggctcagtcgaaagactgggcctttcgttt
tatctgttgtttgtcggtgaacgctctcctgagtaggacaaatccgccgccctagacctaggcgttcggctgcggcgagcggtatcagctcactcaaaggcggtaatacggtt
atccacagaatcaggggataacgcaggaaagaacatgtgagcaaaaggccagcaaaaggccaggaaccgtaaaaaggccgcgttgctggcgttagaatatgtgataca
ggatatattccgcttcctcgctcactgactcgctacgctcggtcgttcgactgcggcgagcggaaatggcttacgaacggggcggagatttcctggaagatgccaggaagat
acttaacagggaagtgagagggccgcggcaaagccgtttttccataggctccgcccccctgacaagcatcacgaaatctgacgctcaaatcagtggtggcgaaacccgac

aggactataaagataccaggcgtttccccctggcggctccctcgtgcgctctcctgttcctgcctttcggtttaccggtgtcattccgctgttatggccgcgtttgtctcattccacg
cctgacactcagttccgggtaggcagttcgctccaagctggactgtatgcacgaacccccgttcagtccgaccgctgcgccttatccggtaactatcgtcttgagtccaacc
cggaaagacatgcaaaagcaccactggcagcagccactggtaattgatttagaggagttagtcttgaagtcatgcgccggttaaggctaaactgaaaggacaagttttggtg
actgcgctcctccaagccagttacctcggttcaaagagttggtagctcagagaaccttcgaaaaaccgccctgcaaggcggttttttcgttttcagagcaagagattacgcgc
agaccaaaacgatctcaagaagatcatcttattaatcagataaaatatttctagatttcagtgcaatttatctcttcaaatgtagcacctgaagtcagccccatacgatataagttgt
aattctcatgttagtcatgccccgcgcccaccggaaggagctgactgggttgaaggctctcaagggcatcggtcgagatcccggtgcctaatgagtgagctaacttacatta
attgcgttgcgcgagctgaagcacacggtcacactgcttccggtagtcaataaaccggtaaaccagcaatagacataagcggctatttaacgaccctgccctgaaccgacg
accgggtcgaatttgctttcgaatttctgccattcatccgcttattatcacttattcaggcgtagcaccaggcgtttaagggcaccaataactgccttaaaaaaattacgccccgc
cctgccactcatcgcagtactgttgtaattcattaagcattctgccgacatggaagccatcacagacggcatgatgaacctgaatcgccagcggcatcagcaccttgtcgcctt
gcgtataatatttgcccatagtgaaaacgggggcgaagaagttgtccatattggccacgtttaaatcaaaactggtgaaactcacccagggattggctgagacgaaaaacat
attctcaataaaccctttagggaaataggccaggttttcaccgtaacacgccacatcttgcgaatatatgtgtagaaactgccggaaatcgtcgtggtattcactccagagcgat
gaaaacgtttcagtttgctcatggaaaacggtgtaacaagggtgaacactatcccatatcaccagctcaccgtctttcattgccatacggaactccggatgagcattcatcagg
cgggcaagaatgtgaataaaggccggataaaacttgtgcttatttttctttacggtctttaaaaaggccgtaatatccagctgaacggtctggttataggtacattgagcaactga
ctgaaatgcctcaaaatgttctttacgatgccattgggatatatcaacggtggtatatccagtgatttttttctccattttagcttccttagctcctgaaaatctcgataactcaaaaaa
tacgcccggtagtgatcttatttcattatggtgaaagttggaacctcttacgtgccgatcaacgtctcattttcgccagatatcgacgtctaagaaaccattattatcatgacattaa
cctataaaaataggcgtatcacgaggccctttcgtcttcac

Appendix Figure S3 | pSClacZ sequence and map

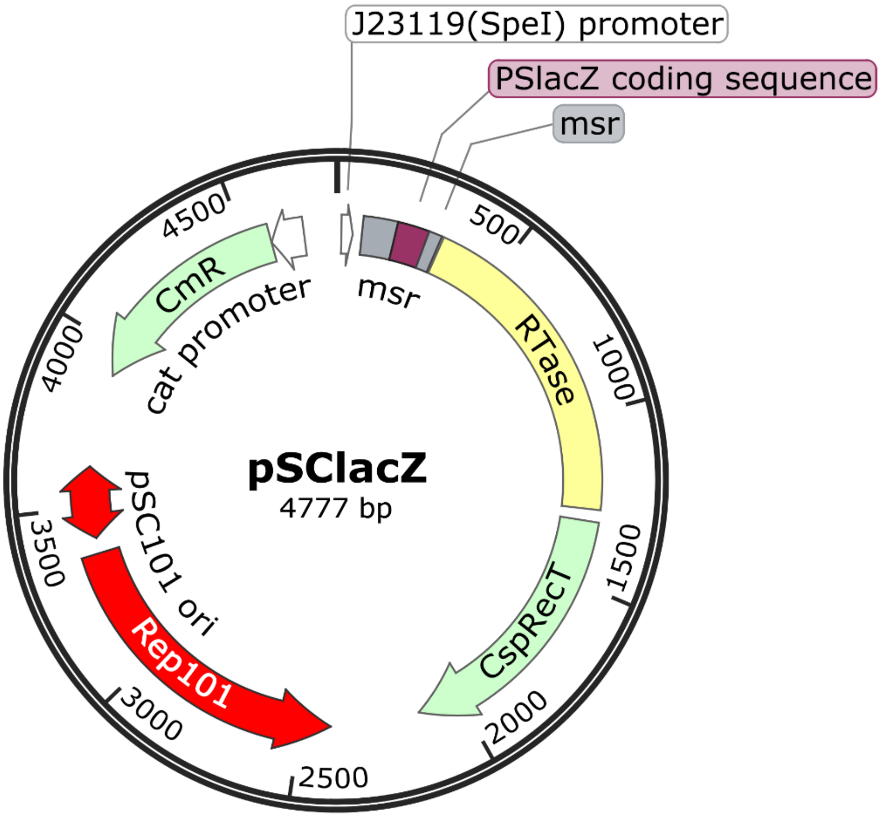

ctcgagatgctagcaattgacagctagctcagtcctaggtataatactagtatcagcaggacgcactgaccttaattaaatgcgcacccttagcgagaggtttatcattaaggtc
aacctctggatgttgtttcggcatcctgcattgaatctgagttactgtctgttttcctgaattcggggatgtgctgcaaggcgattaagttgggtaacgccagggttttatcagtcac
gacgttgtaaaacgacggccagtgaatccgtaatcatggaattcaggaaacccgtttttctgacgtaagggtgcgcaactttcatgaaatccgctgaatatttgaacacttttag
attgagaaatctcggcctacctgtcatgaacaatttgcatgacatgtctaaggcgactcgcatatctgttgaaacacttcggttgttaatctatacagctgattttcgctataggatc
tacactgtagaaaagaaaggcccagagaagagaatgagaaccatttaccaaccttctcgagaacttaaagccttacaaggatgggttctacgtaacattttagataaactgtc
gtcatctcctttttctattggatttgaaaagcaccaatctattttgaataatgctaccccgcatattggggcaaactttatactgaatattgatttggaggatttttttcccaagtttaactg
ctaacaaagtttttggagtgttccattctcttggttataatcgactaatatcttcagttttgacaaaaatatgttgttataaaaatctgctaccacaaggtgctccatcatcacctaaatt
agctaatctaatatgttctaaacttgattatcgtattcagggttatgcaggtagtcggggcttgatatatacgagatatgccgatgacctcaccttatctgcacagtctatgaaaaa
ggttgttaaagcacgtgatttttttattttctataatcccaagtgaaggattggttattaactcaaaaaaaacttgtattagtgggcctcgtagtcagaggaaagttacaggtttagtta
tttcacaagagaaagttgggataggtagagaaaaatataaagaaattagagcaaagatacatcatatattttgcggtaagtcttctgagatagaacacgttaggggatggttgtc
atttattttaagtgtggattcaaaaagccataggagattaataacttatattagcaaattagaaaaaaaatatggaaagaaccctttaaataaagcgaagacctaaggatccggtt
gatattgattcagaggtataaaacgaatgaaccaaatcgtgaagttcactgacgactctggcctggcggttcaagttactccagacgatgttcgccgttatatctgtgagaacg
ctactgaaaaagaggtgggcctctttctgcaactctgtcagactcaacgtctgaatccgtttgtgaaagacgcttacctggtgaaatacggcggtgctccagcttctatgattact
tcctatcaagtttttaaccgtcgcgcgtgtcgtgatgctaactatgatggtatcaaatctggtgtggttgttctgcgtgacggtgatgttgtgcataaacgtggtgctgcgtgctac
aaaaaggcgggtgaggagctcatcggtggttgggcggaagttcgctttaaggatggccgcgagactgcgtatgctgaggtggcgctcgacgactattccaccggcaaatc
taattgggcgaaaatgccgggtgttatgatcgaaaaatgcgcgaaggctgctgcttggcgcctcgcgttcccggacacttttcagggcatgtacgctgcggaggaaatggat
caagcgcaacagccagaacaggtgcgcgctcaggcggagcaaccagtggatctccagccaatccgcgaactcttcaagccatattgcgaacacttcggcatcactccgg
ctgagggtatgactgctgtttgtggtgcggtgggcgctgaaggcatgcactctatgaccgagcagcaagctcgccgtgctcgcgcttggatggaggaagaaatggctgcg
ccagctgtggaagcggagtatgaggttgttgacgagggcgaggtgttttaaacgcgtgctagaggcatcaaataaaacgaaaggctcagtcgaaagactgggcctttcgttt
tatctgttgtttgtcggtgaacgctctcctgagtaggacaaatccgccgccctagacctaggcgttcggctgcggcgaggaaaagatcaaaggatcttccgttttcatctgtgc
atatggacagttttccctttgatatctaacggtgaacagttgttctacttttgtttgttagtcttgatgcttcactgatagatacaagagccataagaacctcagatccttccgtatttag
ccagtatgttctctagtgtggttcgttgtttttgcgtgagccatgagaacgaaccattgagatcatgcttactttgcatgtcactcaaaaattttgcctcaaaactggtgagctgaatt
tttgcagttaaagcatcgtgtagtgtttttcttagtccgttacgtaggtaggaatctgatgtaatggttgttggtattttgtcaccattcatttttatctggttgttctcaagttcggttacg

agatccatttgtctatctagttcaacttggaaaatcaacgtatcagtcgggcggcctcgcttatcaaccaccaatttcatattgctgtaagtgtttaaatctttacttattggtttcaaa
acccattggttaagccttttaaactcatggtagttattttcaagcattaacatgaacttaaattcatcaaggctaatctctatatttgccttgtgagttttcttttgtgttagttcttttaataa
ccactcataaatcctcatagagtatttgttttcaaaagacttaacatgttccagattatattttatgaatttttttaactggaaaagataaggcaatatctcttcactaaaaactaattcta
atttttcgcttgagaacttggcatagtttgtccactggaaaatctcaaagcctttaaccaaaggattcctgatttccacagttctcgtcatcagctctctggttgctttagctaataca
ccataagcattttccctactgatgttcatcatctgagcgtattggttataagtgaacgataccgtccgttctttccttgtagggttttcaatcgtggggttgagtagtgccacacagc
ataaaattagcttggtttcatgctccgttaagtcatagcgactaatcgctagttcatttgctttgaaaacaactaattcagacatacatctcaattggtctaggtgattttaatcactat
accaattgagatgggctagtcaatgataattactagtccttttcctttgagttgtgggtatctgtaaattctgctagacctttgctggaaaacttgtaaattctgctagaccctctgtaa
attccgctagacctttgtgtgttttttttgtttatattcaagtggttataatttatagaataaagaaagaataaaaaaagataaaaagaatagatcccagccctgtgtataactcactac
tttagtcagttccgcagtattacaaaaggatgtcgcaaacgctgtttgctcctctacaaaacagaccttaaaaccctaaaggcttaagtagcaccatctgtgcggtatttcacacc
gctcagtggaacgaaaactcacgttaagggattttggtcatgactagtgcttggattctcaccaataaaaaacgcccggcggcaaccgagcgttctgaacaaatccagatgg
agttctgaggtcattactggatctatcaacaggagtccaagcgagctcgatatcaaattacgccccgccctgccactcatcgcagtactgttgtaattcattaagcattctgccg
acatggaagccatcacagacggcatgatgaacctgaatcgccagcggcatcagcaccttgtcgccttgcgtataatatttgcccatagtgaaaacgggggcgaagaagttg
tccatattggccacgtttaaatcaaaactggtgaaactcacccagggattggctgagacgaaaaacatattctcaataaaccctttagggaaataggccaggttttcaccgtaa
cacgccacatcttgcgaatatatgtgtagaaactgccggaaatcgtcgtggtattcactccagagcgatgaaaacgtttcagtttgctcatggaaaacggtgtaacaagggtg
aacactatcccatatcaccagctcaccgtctttcattgccatacggaactccggatgagcattcatcaggcgggcaagaatgtgaataaaggccggataaaacttgtgcttattt
ttctttacggtctttaaaaaggccgtaatatccagctgaacggtctggttataggtacattgagcaactgactgaaatgcctcaaaatgttctttacgatgccattgggatatatca
acggtggtatatccagtgatttttttctccattttagcttccttagctcctgaaaatctcgataactcaaaaaatacgcccggtagtgatcttatttcattatggtgaaagttggaacct
cttacgtgccgatcaacgtctcattttcgccagatatcgacgtctaagaaaccattattatcatgacattaacctataaaaataggcgtatcacgaggccctttcgtcttcac

Appendix Figure S4 | pUClacZ sequence and map

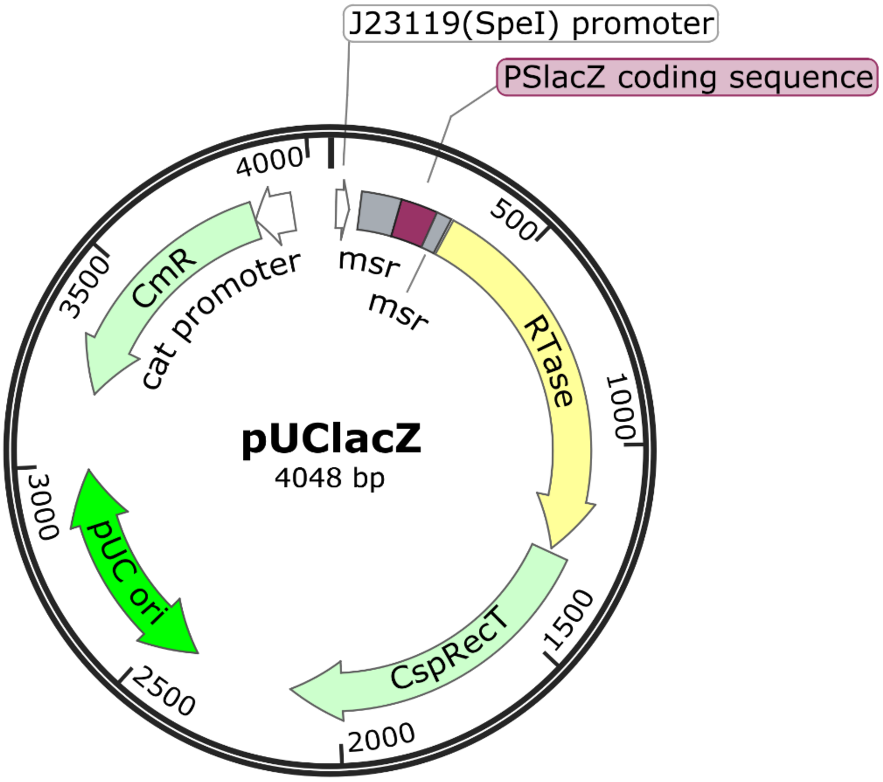

ctcgagatgctagcaattgacagctagctcagtcctaggtataatactagtatcagcaggacgcactgaccttaattaaatgcgcacccttagcgagaggtttatcattaaggtc
aacctctggatgttgtttcggcatcctgcattgaatctgagttactgtctgttttcctgaattcggggatgtgctgcaaggcgattaagttgggtaacgccagggttttatcagtcac
gacgttgtaaaacgacggccagtgaatccgtaatcatggaattcaggaaacccgtttttctgacgtaagggtgcgcaactttcatgaaatccgctgaatatttgaacacttttag
attgagaaatctcggcctacctgtcatgaacaatttgcatgacatgtctaaggcgactcgcatatctgttgaaacacttcggttgttaatctatacagctgattttcgctataggatc
tacactgtagaaaagaaaggcccagagaagagaatgagaaccatttaccaaccttctcgagaacttaaagccttacaaggatgggttctacgtaacattttagataaactgtc
gtcatctccttttctattggatttgaaaagcaccaatctattttgaataatgctaccccgcatattggggcaaactttatactgaatattgatttggaggatttttcccaagtttaactg
ctaacaaagtttttggagtgttccattctcttggttataatcgactaatatcttcagttttgacaaaaatatgttgttataaaaatctgctaccacaaggtgctccatcatcacctaaatt
agctaatctaatatgttctaaacttgattatcgtattcagggttatgcaggtagtcggggcttgatatatacgagatatgccgatgacctcaccttatctgcacagtctatgaaaaa
ggttgttaaagcacgtgatttttttattttctataatcccaagtgaaggattggttattaactcaaaaaaaacttgtattagtgggcctcgtagtcagaggaaagttacaggtttagtta
tttcacaagagaaagttgggataggtagagaaaaatataaagaaattagagcaaagatacatcatatattttgcggtaagtcttctgagatagaacacgttaggggatggttgtc
atttattttaagtgtggattcaaaaagccataggagattaataacttatattagcaaattagaaaaaaaatatggaaagaacccttttaaataaagcgaagacctaaggatccggtt
gatattgattcagaggtataaaacgaatgaaccaaatcgtgaagttcactgacgactctggcctggcggttcaagttactccagacgatgttcgccgttatatctgtgagaacg
ctactgaaaaagaggtgggcctctttctgcaactctgtcagactcaacgtctgaatccgtttgtgaaagacgcttacctggtgaaatacggcggtgctccagcttctatgattact
tcctatcaagtttttaaccgtcgcgcgtgtcgtgatgctaactatgatggtatcaaatctggtgtggttgttctgcgtgacggtgatgttgtgcataaacgtggtgctgcgtgctac
aaaaaggcgggtgaggagctcatcggtggttgggcggaagttcgctttaaggatggccgcgagactgcgtatgctgaggtggcgctcgacgactattccaccggcaaatc
taattgggcgaaaatgccgggtgttatgatcgaaaaatgcgcgaaggctgctgcttggcgcctcgcgttcccggacacttttcagggcatgtacgctgcggaggaaatggat
caagcgcaacagccagaacaggtgcgcgctcaggcggagcaaccagtggatctccagccaatccgcgaactcttcaagccatattgcgaacacttcggcatcactccgg
ctgagggtatgactgctgtttgtggtgcggtgggcgctgaaggcatgcactctatgaccgagcagcaagctcgccgtgctcgcgcttggatggaggaagaaatggctgcg
ccagctgtggaagcggagtatgaggttgttgacgagggcgaggtgttttaaacgcgtgctagaggcatcaaataaaacgaaaggctcagtcgaaagactgggcctttcgttt
tatctgttgtttgtcggtgaacgctctcctgagtaggacaaatccgccgccctagacctaggcgttcggctgcggcgagcggtatcagctcactcaaaggcggtaatacggtt
atccacagaatcaggggataacgcaggaaagaacatgtgagcaaaaggccagcaaaaggccaggaaccgtaaaaaggccgcgttgctggcgtttttccataggctccgc
ccccctgacgagcatcacaaaaatcgacgctcaagtcagaggtggcgaaacccgacaggactataaagataccaggcgtttccccctggaagctccctcgtgcgctctcct
gttccgaccctgccgcttaccggatacctgtccgcctttctcccttcgggaagcgtggcgctttctcatagctcacgctgtaggtatctcagttcggtgtaggtcgttcgctccaa
gctgggctgtgtgcacgaaccccccgttcagcccgaccgctgcgccttatccggtaactatcgtcttgagtccaacccggtaagacacgacttatcgccactggcagcagc

cactggtaacaggattagcagagcgaggtatgtaggcggtgctacagagttcttgaagtggtggcctaactacggctacactagaagAacagtatttggtatctgcgctctg
ctgaagccagttaccttcggaaaaagagttggtagctcttgatccggcaaacaaaccaccgctggtagcggtggtttttttgtttgcaagcagcagattacgcgcagaaaaaa
aggatctcaagaagatcctttgatcttttctacggggtctgacgctcagtggaacgaaaactcacgttaagggattttggtcatgactagtgcttggattctcaccaataaaaaac
gcccggcggcaaccgagcgttctgaacaaatccagatggagttctgaggtcattactggatctatcaacaggagtccaagcgagctcgatatcaaattacgccccgccctg
ccactcatcgcagtactgttgtaattcattaagcattctgccgacatggaagccatcacagacggcatgatgaacctgaatcgccagcggcatcagcaccttgtcgccttgcgt
ataatatttgcccatggtgaaaacgggggcgaagaagttgtccatattggccacgtttaaatcaaaactggtgaaactcacccagggattggctgagacgaaaaacatattct
caataaaccctttagggaaataggccaggttttcaccgtaacacgccacatcttgcgaatatatgtgtagaaactgccggaaatcgtcgtggtattcactccagagcgatgaaa
acgtttcagtttgctcatggaaaacggtgtaacaagggtgaacactatcccatatcaccagctcaccgtctttcattgccatacggaattccggatgagcattcatcaggcggg
caagaatgtgaataaaggccggataaaacttgtgcttatttttctttacggtctttaaaaaggccgtaatatccagctgaacggtctggttataggtacattgagcaactgactga
aatgcctcaaaatgttctttacgatgccattgggatatatcaacggtggtatatccagtgatttttttctccattttagcttccttagctcctgaaaatctcgataactcaaaaaatacg
cccggtagtgatcttatttcattatggtgaaagttggaacctcttacgtgccgatcaacgtctcattttcgccagatatcgacgtctaagaaaccattattatcatgacattaacctat
aaaaataggcgtatcacgaggccctttcgtcttcac

Appendix Figure S5 | p15AlacZ-lacZ sequence and map

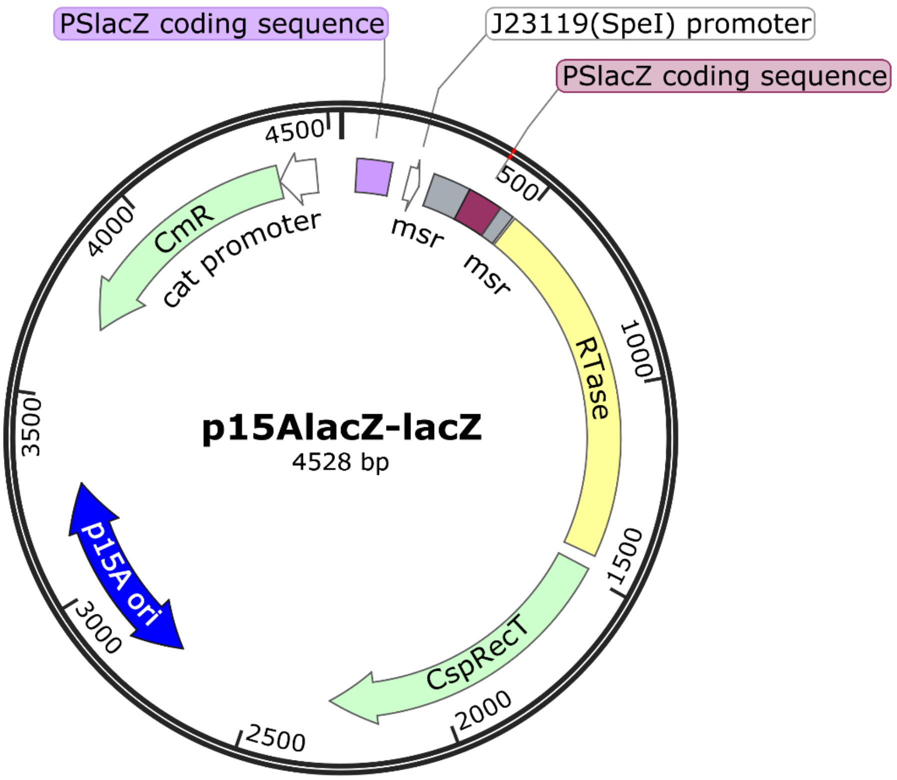

aataggcgtatcacgaggcccttcggtatttcacaccgcataggggatgtgctgcaaggcgattaagttgggtaacgccagggttttcccagtcacgacgttgtaaaacgac
ggccagtgaatccgtaatcatggtcagcggtggagtgcaatgtctcgtcttcacctcgagatgctagcaattgacagctagctcagtcctaggtataatactagtatcagcagg
acgcactgaccttaattaaatgcgcacccttagcgagaggtttatcattaaggtcaacctctggatgttgtttcggcatcctgcattgaatctgagttactgtctgttttcctgaattc
ggggatgtgctgcaaggcgattaagttgggtaacgccagggttttatcagtcacgacgttgtaaaacgacggccagtgaatccgtaatcatggaattcaggaaacccgtttttt
ctgacgtaagggtgcgcaactttcatgaaatccgctgaatatttgaacacttttagattgagaaatctcggcctacctgtcatgaacaatttgcatgacatgtctaaggcgactcg
catatctgttgaaacacttcggttgttaatctatacagctgattttcgctataggatctacactgtagaaaagaaaggcccagagaagagaatgagaaccatttaccaaccttctc
gagaacttaaagccttacaaggatgggttctacgtaacattttagataaactgtcgtcatctcctttttctattggatttgaaaagcaccaatctattttgaataatgctaccccgcat
attggggcaaactttatactgaatattgatttggaggatttttttcccaagtttaactgctaacaaagtttttggagtgttccattctcttggttataatcgactaatatcttcagttttgaca
aaaatatgttgttataaaaatctgctaccacaaggtgctccatcatcacctaaattagctaatctaatatgttctaaacttgattatcgtattcagggttatgcaggtagtcggggctt
gatatatacgagatatgccgatgacctcaccttatctgcacagtctatgaaaaaggttgttaaagcacgtgatttttttattttctataatcccaagtgaaggattggttattaactcaa
aaaaaacttgtattagtgggcctcgtagtcagaggaaagttacaggtttagttatttcacaagagaaagttgggataggtagagaaaaatataaagaaattagagcaaagatac
atcatatattttgcggtaagtcttctgagatagaacacgttaggggatggttgtcatttattttaagtgtggattcaaaaagccataggagattaataacttatattagcaaattagaa
aaaaaatatggaaagaaccctttaaataaagcgaagacctaaggatccggttgatattgattcagaggtataaaacgaatgaaccaaatcgtgaagttcactgacgactctgg
cctggcggttcaagttactccagacgatgttcgccgttatatctgtgagaacgctactgaaaaagaggtgggcctctttctgcaactctgtcagactcaacgtctgaatccgttt
gtgaaagacgcttacctggtgaaatacggcggtgctccagcttctatgattacttcctatcaagtttttaaccgtcgcgcgtgtcgtgatgctaactatgatggtatcaaatctggt
gtggttgttctgcgtgacggtgatgttgtgcataaacgtggtgctgcgtgctacaaaaaggcgggtgaggagctcatcggtggttgggcggaagttcgctttaaggatggcc
gcgagactgcgtatgctgaggtggcgctcgacgactattccaccggcaaatctaattgggcgaaaatgccgggtgttatgatcgaaaaatgcgcgaaggctgctgcttggc
gcctcgcgttcccggacacttttcagggcatgtacgctgcggaggaaatggatcaagcgcaacagccagaacaggtgcgcgctcaggcggagcaaccagtggatctcca
gccaatccgcgaactcttcaagccatattgcgaacacttcggcatcactccggctgagggtatgactgctgtttgtggtgcggtgggcgctgaaggcatgcactctatgaccg
agcagcaagctcgccgtgctcgcgcttggatggaggaagaaatggctgcgccagctgtggaagcggagtatgaggttgttgacgagggcgaggtgttttaaacgcgtgct
agaggcatcaaataaaacgaaaggctcagtcgaaagactgggcctttcgttttatctgttgtttgtcggtgaacgctctcctgagtaggacaaatccgccgccctagacctag
gcgttcggctgcggcgagcggtatcagctcactcaaaggcggtaatacggttatccacagaatcaggggataacgcaggaaagaacatgtgagcaaaaggccagcaaa
aggccaggaaccgtaaaaaggccgcgttgctggcgttagaatatgtgatacaggatatattccgcttcctcgctcactgactcgctacgctcggtcgttcgactgcggcgag
cggaaatggcttacgaacggggcggagatttcctggaagatgccaggaagatacttaacagggaagtgagagggccgcggcaaagccgtttttccataggctccgcccc

cctgacaagcatcacgaaatctgacgctcaaatcagtggtggcgaaacccgacaggactataaagataccaggcgtttccccctggcggctccctcgtgcgctctcctgttcc
tgcctttcggtttaccggtgtcattccgctgttatggccgcgtttgtctcattccacgcctgacactcagttccgggtaggcagttcgctccaagctggactgtatgcacgaaccc
cccgttcagtccgaccgctgcgccttatccggtaactatcgtcttgagtccaacccggaaagacatgcaaaagcaccactggcagcagccactggtaattgatttagaggag
ttagtcttgaagtcatgcgccggttaaggctaaactgaaaggacaagttttggtgactgcgctcctccaagccagttacctcggttcaaagagttggtagctcagagaaccttc
gaaaaaccgccctgcaaggcggttttttcgttttcagagcaagagattacgcgcagaccaaaacgatctcaagaagatcatcttattaatcagataaaatatttctagatttcagt
gcaatttatctcttcaaatgtagcacctgaagtcagccccatacgatataagttgtaattctcatgttagtcatgccccgcgcccaccggaaggagctgactgggttgaaggctc
tcaagggcatcggtcgagatcccggtgcctaatgagtgagctaacttacattaattgcgttgcgcgagctgaagcacacggtcacactgcttccggtagtcaataaaccggta
aaccagcaatagacataagcggctatttaacgaccctgccctgaaccgacgaccgggtcgaatttgctttcgaatttctgccattcatccgcttattatcacttattcaggcgtag
caccaggcgtttaagggcaccaataactgccttaaaaaaattacgccccgccctgccactcatcgcagtactgttgtaattcattaagcattctgccgacatggaagccatcac
agacggcatgatgaacctgaatcgccagcggcatcagcaccttgtcgccttgcgtataatatttgcccatagtgaaaacgggggcgaagaagttgtccatattggccacgttt
aaatcaaaactggtgaaactcacccagggattggctgagacgaaaaacatattctcaataaaccctttagggaaataggccaggttttcaccgtaacacgccacatcttgcga
atatatgtgtagaaactgccggaaatcgtcgtggtattcactccagagcgatgaaaacgtttcagtttgctcatggaaaacggtgtaacaagggtgaacactatcccatatcac
cagctcaccgtctttcattgccatacggaactccggatgagcattcatcaggcgggcaagaatgtgaataaaggccggataaaacttgtgcttatttttctttacggtctttaaaa
aggccgtaatatccagctgaacggtctggttataggtacattgagcaactgactgaaatgcctcaaaatgttctttacgatgccattgggatatatcaacggtggtatatccagt
gatttttttctccattttagcttccttagctcctgaaaatctcgataactcaaaaaatacgcccggtagtgatcttatttcattatggtgaaagttggaacctcttacgtgccgatcaac
gtctcattttcgccagatatcgacgtctaagaaaccattattatcatgacattaacctataaa

Appendix Figure S6 | pBR-PkanX sequence and map

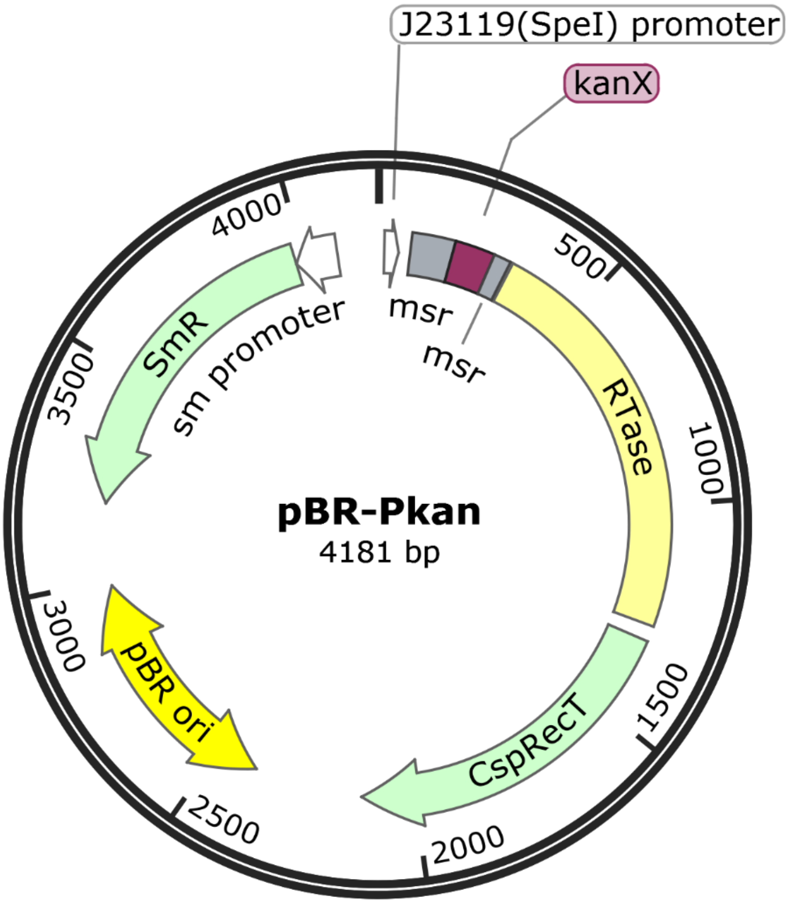

ctcgagatgctagcaattgacagctagctcagtcctaggtataatactagtatcagcaggacgcactgaccttaattaaatgcgcacccttagcgagaggtttatcattaaggtc
aacctctggatgttgtttcggcatcctgcattgaatctgagttactgtctgttttcctgaattcgtcttgctctaggccgcgattaaattccaacatggatgctgatttatatgggtata
aatgggctcgcgataatgtcgggcaatcaggtgcgacgaattcaggaaacccgtttttctgacgtaagggtgcgcaactttcatgaaatccgctgaatatttgaacacttttag
attgagaaatctcggcctacctgtcatgaacaatttgcatgacatgtctaaggcgactcgcatatctgttgaaacacttcggttgttaatctatacagctgattttcgctataggatc
tacactgtagaaaagaaaggcccagagaagagaatgagaaccatttaccaaccttctcgagaacttaaagccttacaaggatgggttctacgtaacattttagataaactgtc
gtcatctcctttttctattggatttgaaaagcaccaatctattttgaataatgctaccccgcatattggggcaaactttatactgaatattgatttggaggatttttcccaagtttaactg
ctaacaaagtttttggagtgttccattctcttggttataatcgactaatatcttcagttttgacaaaaatatgttgttataaaaatctgctaccacaaggtgctccatcatcacctaaatt
agctaatctaatatgttctaaacttgattatcgtattcagggttatgcaggtagtcggggcttgatatatacgagatatgccgatgacctcaccttatctgcacagtctatgaaaaa
ggttgttaaagcacgtgatttttttattttctataatcccaagtgaaggattggttattaactcaaaaaaaacttgtattagtgggcctcgtagtcagaggaaagttacaggtttagtta
tttcacaagagaaagttgggataggtagagaaaaatataaagaaattagagcaaagatacatcatatattttgcggtaagtcttctgagatagaacacgttaggggatggttgtc
atttattttaagtgtggattcaaaaagccataggagattaataacttatattagcaaattagaaaaaaaatatggaaagaaccctttaaataaagcgaagacctaaggatccggtt
gatattgattcagaggtataaaacgaatgaaccaaatcgtgaagttcactgacgactctggcctggcggttcaagttactccagacgatgttcgccgttatatctgtgagaacg
ctactgaaaaagaggtgggcctctttctgcaactctgtcagactcaacgtctgaatccgtttgtgaaagacgcttacctggtgaaatacggcggtgctccagcttctatgattact
tcctatcaagtttttaaccgtcgcgcgtgtcgtgatgctaactatgatggtatcaaatctggtgtggttgttctgcgtgacggtgatgttgtgcataaacgtggtgctgcgtgctac
aaaaaggcgggtgaggagctcatcggtggttgggcggaagttcgctttaaggatggccgcgagactgcgtatgctgaggtggcgctcgacgactattccaccggcaaatc
taattgggcgaaaatgccgggtgttatgatcgaaaaatgcgcgaaggctgctgcttggcgcctcgcgttcccggacacttttcagggcatgtacgctgcggaggaaatggat
caagcgcaacagccagaacaggtgcgcgctcaggcggagcaaccagtggatctccagccaatccgcgaactcttcaagccatattgcgaacacttcggcatcactccgg
ctgagggtatgactgctgtttgtggtgcggtgggcgctgaaggcatgcactctatgaccgagcagcaagctcgccgtgctcgcgcttggatggaggaagaaatggctgcg
ccagctgtggaagcggagtatgaggttgttgacgagggcgaggtgttttaaacgcgtgctagaggcatcaaataaaacgaaaggctcagtcgaaagactgggcctttcgttt
tatctgttgtttgtcggtgaacgctctcctgagtaggacaaatccgccgccctagacctaggcgttcggctgcggcgagcggtatcagctcactcaaaggcggtaatacggtt

atccacagaatcaggggataacgcaggaaagaacatgtgagcaaaaggccagcaaaaggccaggaaccgtaaaaaggccgcgttgctggcgtttttccataggctccgc
cccccctgacgagcatcacaaaaatcgacgctcaagtcagaggtggcgaaacccgacaggactataaagataccaggcgtttccccctggaagctccctcgtgcgctctcct
gttccgaccctgccgcttaccggatacctgtccgcctttctcccttcgggaagcgtggcgctttctcaatgctcacgctgtaggtatctcagttcggtgtaggtcgttcgctccaa
gctgggctgtgtgcacgaaccccccgttcagcccgaccgctgcgccttatccggtaactatcgtcttgagtccaacccggtaagacacgacttatcgccactggcagcagc
cactggtaacaggattagcagagcgaggtatgtaggcggtgctacagagttcttgaagtggtggcctaactacggctacactagaaggacagtatttggtatctgcgctctgc
tgaagccagttaccttcggaaaaagagttggtagctcttgatccggcaaacaaaccaccgctggtagcggtggtttttttgtttgcaagcagcagattacgcgcagaaaaaaa
ggatctcaagaagatcctttgatcttttctacggggtctgacgctcagtggaacgaaaactcacgttaagggattttggtcatgactagtgcttggattctcaccaataaaaaacg
cccggcggcaaccgagcgttctgaacaaatccagatggagttctgaggtcattactggatctatcaacaggagtccaagcgagctcgatatcaaattatttgccgactacctt
ggtgatctcgcctttcacgtagtggacaaattcttccaactgatctgcgcgcgaggccaagcgatcttcttcttgtccaagataagcctgtctagcttcaagtatgacgggctga
tactgggccggcaggcgctccattgcccagtcggcagcgacatccttcggcgcgattttgccggttactgcgctgtaccaaatgcgggacaacgtaagcactacatttcgct
catcgccagcccagtcgggcggcgagttccatagcgttaaggtttcatttagcgcctcaaatagatcctgttcaggaaccggatcaaagagttcctccgccgctggacctac
caaggcaacgctatgttctcttgcttttgtcagcaagatagccagatcaatgtcgatcgtggctggctcgaagatacctgcaagaatgtcattgcgctgccattctccaaattgc
agttcgcgcttagctggataacgccacggaatgatgtcgtcgtgcacaacaatggtgacttctacagcgcggagaatctcgctctctccaggggaagccgaagtttccaaaa
ggtcgttgatcaaagctcgccgcgttgtttcatcaagccttacggtcaccgtaaccagcaaatcaatatcactgtgtggcttcaggccgccatccactgcggagccgtacaaa
tgtacggccagcaacgtcggttcgagatggcgctcgatgacgccaactacctctgatagttgagtcgatacttcggcgatcaccgcttccctcattttagcttccttagctcctg
aaaatctcgataactcaaaaaatacgcccggtagtgatcttatttcattatggtgaaagttggaacctcttacgtgccgatcaacgtctcattttcgccagatatcgacgtctaaga
aaccattattatcatgacattaacctataaaaataggcgtatcacgaggccctttcgtcttcac

Appendix Figure S7 | p15A- PkanY sequence and map

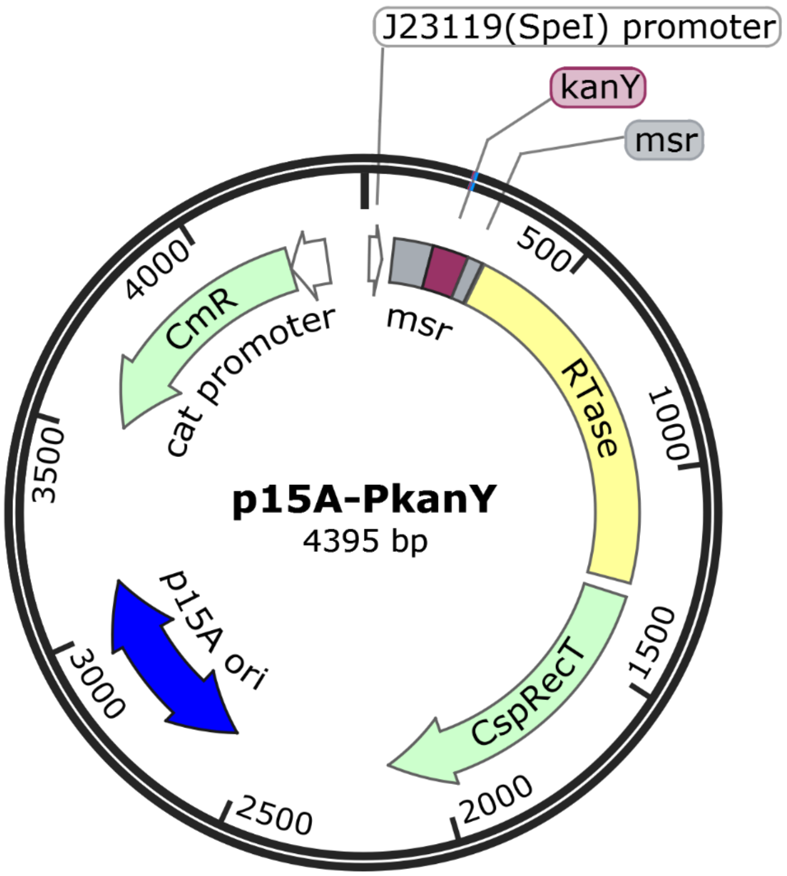

ctcgagatgctagcaattgacagctagctcagtcctaggtataatactagtatcagcaggacgcactgaccttaattaaatgcgcacccttagcgagaggtttatcattaaggtc
aacctctggatgttgtttcggcatcctgcattgaatctgagttactgtctgttttcctgaattcgtcttgctctaggccgcgattaaattccaacatggatgctgattgataagggtat
aaatgggctcgcgataatgtcgggcaatcaggtgcgacaattcaggaaacccgtttttctgacgtaagggtgcgcaactttcatgaaatccgctgaatatttgaacacttttag
attgagaaatctcggcctacctgtcatgaacaatttgcatgacatgtctaaggcgactcgcatatctgttgaaacacttcggttgttaatctatacagctgattttcgctataggatc
tacactgtagaaaagaaaggcccagagaagagaatgagaaccatttaccaaccttctcgagaacttaaagccttacaaggatgggttctacgtaacattttagataaactgtc
gtcatctcctttttctattggatttgaaaagcaccaatctattttgaataatgctaccccgcatattggggcaaactttatactgaatattgatttggaggatttttttcccaagtttaactg
ctaacaaagtttttggagtgttccattctcttggttataatcgactaatatcttcagttttgacaaaaatatgttgttataaaaatctgctaccacaaggtgctccatcatcacctaaatt
agctaatctaatatgttctaaacttgattatcgtattcagggttatgcaggtagtcggggcttgatatatacgagatatgccgatgacctcaccttatctgcacagtctatgaaaaa
ggttgttaaagcacgtgatttttttattttctataatcccaagtgaaggattggttattaactcaaaaaaaacttgtattagtgggcctcgtagtcagaggaaagttacaggtttagtta
tttcacaagagaaagttgggataggtagagaaaaatataaagaaattagagcaaagatacatcatatattttgcggtaagtcttctgagatagaacacgttaggggatggttgtc
atttattttaagtgtggattcaaaaagccataggagattaataacttatattagcaaattagaaaaaaaaatatggaaagaaccctttaaataaagcgaagacctaaggatccggtt
gatattgattcagaggtataaaacgaatgaaccaaatcgtgaagttcactgacgactctggcctggcggttcaagttactccagacgatgttcgccgttatatctgtgagaacg
ctactgaaaaagaggtgggcctctttctgcaactctgtcagactcaacgtctgaatccgtttgtgaaagacgcttacctggtgaaatacggcggtgctccagcttctatgattact
tcctatcaagtttttaaccgtcgcgcgtgtcgtgatgctaactatgatggtatcaaatctggtgtggttgttctgcgtgacggtgatgttgtgcataaacgtggtgctgcgtgctac
aaaaaggcgggtgaggagctcatcggtggttgggcggaagttcgctttaaggatggccgcgagactgcgtatgctgaggtggcgctcgacgactattccaccggcaaatc
taattgggcgaaaatgccgggtgttatgatcgaaaaatgcgcgaaggctgctgcttggcgcctcgcgttcccggacacttttcagggcatgtacgctgcggaggaaatggat
caagcgcaacagccagaacaggtgcgcgctcaggcggagcaaccagtggatctccagccaatccgcgaactcttcaagccatattgcgaacacttcggcatcactccgg
ctgagggtatgactgctgtttgtggtgcggtgggcgctgaaggcatgcactctatgaccgagcagcaagctcgccgtgctcgcgcttggatggaggaagaaatggctgcg
ccagctgtggaagcggagtatgaggttgttgacgagggcgaggtgttttaaacgcgtgctagaggcatcaaataaaacgaaaggctcagtcgaaagactgggcctttcgttt
tatctgttgtttgtcggtgaacgctctcctgagtaggacaaatccgccgccctagacctaggcgttcggctgcggcgagcggtatcagctcactcaaaggcggtaatacggtt
atccacagaatcaggggataacgcaggaaagaacatgtgagcaaaaggccagcaaaaggccaggaaccgtaaaaaggccgcgttgctggcgttagaatatgtgataca

ggatatattccgcttcctcgctcactgactcgctacgctcggtcgttcgactgcggcgagcggaaatggcttacgaacggggcggagatttcctggaagatgccaggaagat
acttaacagggaagtgagagggccgcggcaaagccgtttttccataggctccgcccccctgacaagcatcacgaaatctgacgctcaaatcagtggtggcgaaacccgac
aggactataaagataccaggcgtttcccctggcggctccctcgtgcgctctcctgttcctgcctttcggtttaccggtgtcattccgctgttatggccgcgtttgtctcattccacg
cctgacactcagttccgggtaggcagttcgctccaagctggactgtatgcacgaaccccccgttcagtccgaccgctgcgccttatccggtaactatcgtcttgagtccaacc
cggaaagacatgcaaaagcaccactggcagcagccactggtaattgatttagaggagttagtcttgaagtcatgcgccggttaaggctaaactgaaaggacaagttttggtg
actgcgctcctccaagccagttacctcggttcaaagagttggtagctcagagaaccttcgaaaaaccgccctgcaaggcggttttttcgttttcagagcaagagattacgcgc
agaccaaaacgatctcaagaagatcatcttattaatcagataaaatatttctagatttcagtgcaatttatctcttcaaatgtagcacctgaagtcagccccatacgatataagttgt
aattctcatgttagtcatgccccgcgcccaccggaaggagctgactgggttgaaggctctcaagggcatcggtcgagatcccggtgcctaatgagtgagctaacttacatta
attgcgttgcgcgagctgaagcacacggtcacactgcttccggtagtcaataaaccggtaaaccagcaatagacataagcggctatttaacgaccctgccctgaaccgacg
accgggtcgaatttgctttcgaatttctgccattcatccgcttattatcacttattcaggcgtagcaccaggcgtttaagggcaccaataactgccttaaaaaaattacgccccgc
cctgccactcatcgcagtactgttgtaattcattaagcattctgccgacatggaagccatcacagacggcatgatgaacctgaatcgccagcggcatcagcaccttgtcgcctt
gcgtataatatttgcccatagtgaaaacgggggcgaagaagttgtccatattggccacgtttaaatcaaaactggtgaaactcacccagggattggctgagacgaaaaacat
attctcaataaaccctttagggaaataggccaggttttcaccgtaacacgccacatcttgcgaatatatgtgtagaaactgccggaaatcgtcgtggtattcactccagagcgat
gaaaacgtttcagtttgctcatggaaaacggtgtaacaagggtgaacactatcccatatcaccagctcaccgtctttcattgccatacggaactccggatgagcattcatcagg
cgggcaagaatgtgaataaaggccggataaaacttgtgcttatttttctttacggtctttaaaaaggccgtaatatccagctgaacggtctggttataggtacattgagcaactga
ctgaaatgcctcaaaatgttctttacgatgccattgggatatatcaacggtggtatatccagtgatttttttctccattttagcttccttagctcctgaaaatctcgataactcaaaaaa
tacgcccggtagtgatcttatttcattatggtgaaagttggaacctcttacgtgccgatcaacgtctcattttcgccagatatcgacgtctaagaaaccattattatcatgacattaa
cctataaaaataggcgtatcacgaggccctttcgtcttcac

Appendix Figure S8 | pBR-ØkanX sequence and map

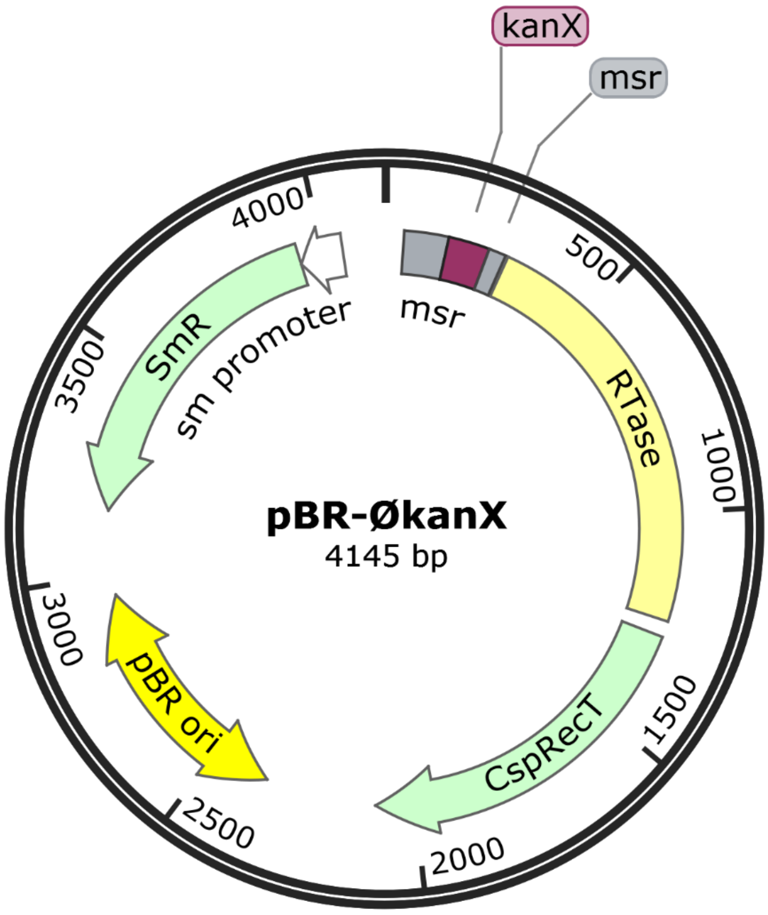

ctcgagatgctagcaaatcagcaggacgcactgaccttaattaaatgcgcacccttagcgagaggtttatcattaaggtcaacctctggatgttgtttcggcatcctgcattgaa
ctcgagatgctagcaaatcagcaggacgcactgaccttaattaaatgcgcacccttagcgagaggtttatcattaaggtcaacctctggatgttgtttcggcatcctgcattgaa
tctgagttactgtctgttttcctgaattcgtcttgctctaggccgcgattaaattccaacatggatgctgatttatatgggtataaatgggctcgcgataatgtcgggcaatcaggtg
cgacaattcaggaaacccgtttttctgacgtaagggtgcgcaactttcatgaaatccgctgaatatttgaacacttttagattgagaaatctcggcctacctgtcatgaacaattt
gcatgacatgtctaaggcgactcgcatatctgttgaaacacttcggttgttaatctatacagctgattttcgctataggatctacactgtagaaaagaaaggcccagagaagaga
atgagaaccatttaccaaccttctcgagaacttaaagccttacaaggatgggttctacgtaacattttagataaactgtcgtcatctcctttttctattggatttgaaaagcaccaatc
tattttgaataatgctaccccgcatattggggcaaactttatactgaatattgatttggaggatttttttcccaagtttaactgctaacaaagtttttggagtgttccattctcttggttata
atcgactaatatcttcagttttgacaaaaatatgttgttataaaaatctgctaccacaaggtgctccatcatcacctaaattagctaatctaatatgttctaaacttgattatcgtattca
gggttatgcaggtagtcggggcttgatatatacgagatatgccgatgacctcaccttatctgcacagtctatgaaaaaggttgttaaagcacgtgatttttttattttctataatccca
agtgaaggattggttattaactcaaaaaaaacttgtattagtgggcctcgtagtcagaggaaagttacaggtttagttatttcacaagagaaagttgggataggtagagaaaaat
ataaagaaattagagcaaagatacatcatatattttgcggtaagtcttctgagatagaacacgttaggggatggttgtcatttattttaagtgtggattcaaaaagccataggagat
taataacttatattagcaaattagaaaaaaaaatatggaaagaaccctttaaataaagcgaagacctaaggatccggttgatattgattcagaggtataaaacgaatgaaccaaat
cgtgaagttcactgacgactctggcctggcggttcaagttactccagacgatgttcgccgttatatctgtgagaacgctactgaaaaagaggtgggcctctttctgcaactctgt
cagactcaacgtctgaatccgtttgtgaaagacgcttacctggtgaaatacggcggtgctccagcttctatgattacttcctatcaagtttttaaccgtcgcgcgtgtcgtgatgct
aactatgatggtatcaaatctggtgtggttgttctgcgtgacggtgatgttgtgcataaacgtggtgctgcgtgctacaaaaaggcgggtgaggagctcatcggtggttgggc
ggaagttcgctttaaggatggccgcgagactgcgtatgctgaggtggcgctcgacgactattccaccggcaaatctaattgggcgaaaatgccgggtgttatgatcgaaaaa
tgcgcgaaggctgctgcttggcgcctcgcgttcccggacacttttcagggcatgtacgctgcggaggaaatggatcaagcgcaacagccagaacaggtgcgcgctcagg
cggagcaaccagtggatctccagccaatccgcgaactcttcaagccatattgcgaacacttcggcatcactccggctgagggtatgactgctgtttgtggtgcggtgggcgc
tgaaggcatgcactctatgaccgagcagcaagctcgccgtgctcgcgcttggatggaggaagaaatggctgcgccagctgtggaagcggagtatgaggttgttgacgag
ggcgaggtgttttaaacgcgtgctagaggcatcaaataaaacgaaaggctcagtcgaaagactgggcctttcgttttatctgttgtttgtcggtgaacgctctcctgagtagga

caaatccgccgccctagacctaggcgttcggctgcggcgagcggtatcagctcactcaaaggcggtaatacggttatccacagaatcaggggataacgcaggaaagaac
atgtgagcaaaaggccagcaaaaggccaggaaccgtaaaaaggccgcgttgctggcgtttttccataggctccgcccccctgacgagcatcacaaaaatcgacgctcaa
gtcagaggtggcgaaacccgacaggactataaagataccaggcgtttccccctggaagctccctcgtgcgctctcctgttccgaccctgccgcttaccggatacctgtccgc
ctttctcccttcgggaagcgtggcgctttctcaatgctcacgctgtaggtatctcagttcggtgtaggtcgttcgctccaagctgggctgtgtgcacgaaccccccgttcagccc
gaccgctgcgccttatccggtaactatcgtcttgagtccaacccggtaagacacgacttatcgccactggcagcagccactggtaacaggattagcagagcgaggtatgta
ggcggtgctacagagttcttgaagtggtggcctaactacggctacactagaaggacagtatttggtatctgcgctctgctgaagccagttaccttcggaaaaagagttggtag
ctcttgatccggcaaacaaaccaccgctggtagcggtggtttttttgtttgcaagcagcagattacgcgcagaaaaaaaggatctcaagaagatcctttgatcttttctacgggg
tctgacgctcagtggaacgaaaactcacgttaagggattttggtcatgactagtgcttggattctcaccaataaaaaacgcccggcggcaaccgagcgttctgaacaaatcca
gatggagttctgaggtcattactggatctatcaacaggagtccaagcgagctcgatatcaaattatttgccgactaccttggtgatctcgcctttcacgtagtggacaaattcttc
caactgatctgcgcgcgaggccaagcgatcttcttcttgtccaagataagcctgtctagcttcaagtatgacgggctgatactgggccggcaggcgctccattgcccagtcg
gcagcgacatccttcggcgcgattttgccggttactgcgctgtaccaaatgcgggacaacgtaagcactacatttcgctcatcgccagcccagtcgggcggcgagttccata
gcgttaaggtttcatttagcgcctcaaatagatcctgttcaggaaccggatcaaagagttcctccgccgctggacctaccaaggcaacgctatgttctcttgcttttgtcagcaa
gatagccagatcaatgtcgatcgtggctggctcgaagatacctgcaagaatgtcattgcgctgccattctccaaattgcagttcgcgcttagctggataacgccacggaatga
tgtcgtcgtgcacaacaatggtgacttctacagcgcggagaatctcgctctctccaggggaagccgaagtttccaaaaggtcgttgatcaaagctcgccgcgttgtttcatca
agccttacggtcaccgtaaccagcaaatcaatatcactgtgtggcttcaggccgccatccactgcggagccgtacaaatgtacggccagcaacgtcggttcgagatggcgc
tcgatgacgccaactacctctgatagttgagtcgatacttcggcgatcaccgcttccctcattttagcttccttagctcctgaaaatctcgataactcaaaaaatacgcccggtag
tgatcttatttcattatggtgaaagttggaacctcttacgtgccgatcaacgtctcattttcgccagatatcgacgtctaagaaaccattattatcatgacattaacctataaaaatag
gcgtatcacgaggccctttcgtcttcac

Appendix Figure S9 | p15A-ØkanY sequence and map

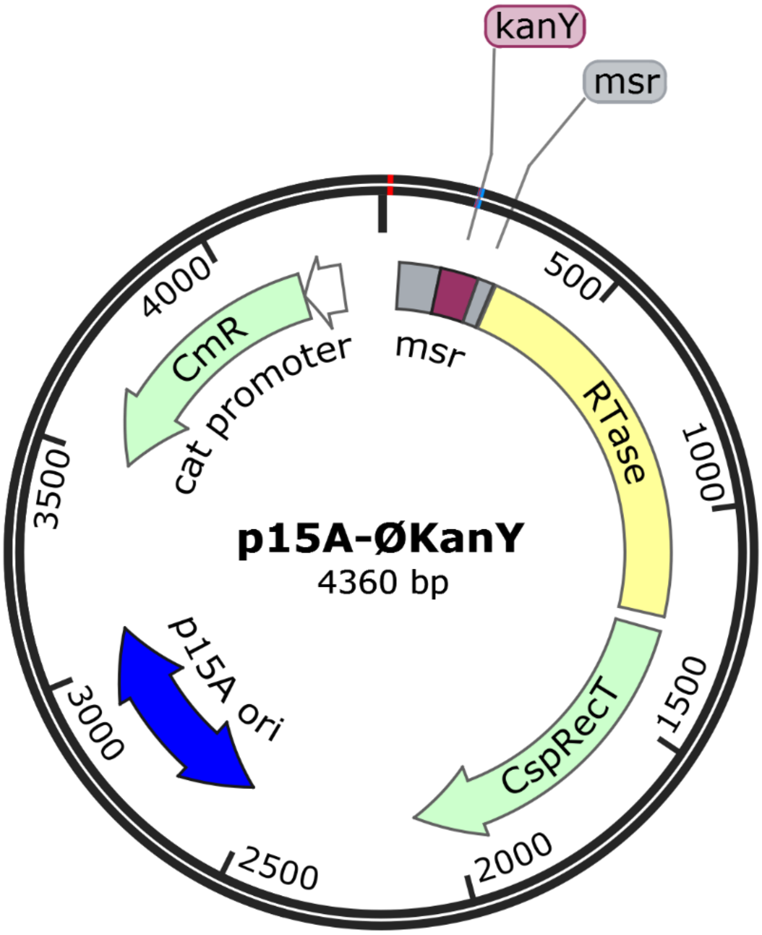

ctcgagatgctagcaaatcagcaggacgcactgaccttaattaaatgcgcacccttagcgagaggtttatcattaaggtcaacctctggatgttgtttcggcatcctgcattgaa
tctgagttactgtctgttttcctgaattcgtcttgctctaggccgcgattaaattccaacatggatgctgattgataagggtataaatgggctcgcgataatgtcgggcaatcaggt
gcgacaattcaggaaacccgtttttctgacgtaagggtgcgcaactttcatgaaatccgctgaatatttgaacacttttagattgagaaatctcggcctacctgtcatgaacaatt
tgcatgacatgtctaaggcgactcgcatatctgttgaaacacttcggttgttaatctatacagctgattttcgctataggatctacactgtagaaaagaaaggcccagagaagag
aatgagaaccatttaccaaccttctcgagaacttaaagccttacaaggatgggttctacgtaacattttagataaactgtcgtcatctcctttttctattggatttgaaaagcaccaat
ctattttgaataatgctaccccgcatattggggcaaactttatactgaatattgatttggaggatttttcccaagtttaactgctaacaaagtttttggagtgttccattctcttggttat
aatcgactaatatcttcagttttgacaaaaatatgttgttataaaaatctgctaccacaaggtgctccatcatcacctaaattagctaatctaatatgttctaaacttgattatcgtattc
agggttatgcaggtagtcggggcttgatatatacgagatatgccgatgacctcaccttatctgcacagtctatgaaaaaggttgttaaagcacgtgatttttttattttctataatccc
aagtgaaggattggttattaactcaaaaaaaacttgtattagtgggcctcgtagtcagaggaaagttacaggtttagttatttcacaagagaaagttgggataggtagagaaaa
atataaagaaattagagcaaagatacatcatatattttgcggtaagtcttctgagatagaacacgttaggggatggttgtcatttattttaagtgtggattcaaaaagccataggag
attaataacttatattagcaaattagaaaaaaaatatggaaagaaccctttaaataaagcgaagacctaaggatccggttgatattgattcagaggtataaaacgaatgaaccaa
atcgtgaagttcactgacgactctggcctggcggttcaagttactccagacgatgttcgccgttatatctgtgagaacgctactgaaaaagaggtgggcctctttctgcaactct
gtcagactcaacgtctgaatccgtttgtgaaagacgcttacctggtgaaatacggcggtgctccagcttctatgattacttcctatcaagtttttaaccgtcgcgcgtgtcgtgatg
ctaactatgatggtatcaaatctggtgtggttgttctgcgtgacggtgatgttgtgcataaacgtggtgctgcgtgctacaaaaaggcgggtgaggagctcatcggtggttggg
cggaagttcgctttaaggatggccgcgagactgcgtatgctgaggtggcgctcgacgactattccaccggcaaatctaattgggcgaaaatgccgggtgttatgatcgaaaa
atgcgcgaaggctgctgcttggcgcctcgcgttcccggacacttttcagggcatgtacgctgcggaggaaatggatcaagcgcaacagccagaacaggtgcgcgctcag
gcggagcaaccagtggatctccagccaatccgcgaactcttcaagccatattgcgaacacttcggcatcactccggctgagggtatgactgctgtttgtggtgcggtgggcg
ctgaaggcatgcactctatgaccgagcagcaagctcgccgtgctcgcgcttggatggaggaagaaatggctgcgccagctgtggaagcggagtatgaggttgttgacga
gggcgaggtgttttaaacgcgtgctagaggcatcaaataaaacgaaaggctcagtcgaaagactgggcctttcgttttatctgttgtttgtcggtgaacgctctcctgagtagg

acaaatccgccgccctagacctaggcgttcggctgcggcgagcggtatcagctcactcaaaggcggtaatacggttatccacagaatcaggggataacgcaggaaagaa
catgtgagcaaaaggccagcaaaaggccaggaaccgtaaaaaggccgcgttgctggcgttagaatatgtgatacaggatatattccgcttcctcgctcactgactcgctacg
ctcggtcgttcgactgcggcgagcggaaatggcttacgaacggggcggagatttcctggaagatgccaggaagatacttaacagggaagtgagagggccgcggcaaag
ccgtttttccataggctccgccccccctgacaagcatcacgaaatctgacgctcaaatcagtggtggcgaaacccgacaggactataaagataccaggcgtttccccctggcgg
ctccctcgtgcgctctcctgttcctgcctttcggtttaccggtgtcattccgctgttatggccgcgtttgtctcattccacgcctgacactcagttccgggtaggcagttcgctcca
agctggactgtatgcacgaaccccccgttcagtccgaccgctgcgccttatccggtaactatcgtcttgagtccaacccggaaagacatgcaaaagcaccactggcagcag
ccactggtaattgatttagaggagttagtcttgaagtcatgcgccggttaaggctaaactgaaaggacaagttttggtgactgcgctcctccaagccagttacctcggttcaaa
gagttggtagctcagagaaccttcgaaaaaccgccctgcaaggcggttttttcgttttcagagcaagagattacgcgcagaccaaaacgatctcaagaagatcatcttattaat
cagataaaatatttctagatttcagtgcaatttatctcttcaaatgtagcacctgaagtcagccccatacgatataagttgtaattctcatgttagtcatgccccgcgcccaccgga
aggagctgactgggttgaaggctctcaagggcatcggtcgagatcccggtgcctaatgagtgagctaacttacattaattgcgttgcgcgagctgaagcacacggtcacact
gcttccggtagtcaataaaccggtaaaccagcaatagacataagcggctatttaacgaccctgccctgaaccgacgaccgggtcgaatttgctttcgaatttctgccattcatc
cgcttattatcacttattcaggcgtagcaccaggcgtttaagggcaccaataactgccttaaaaaaattacgccccgccctgccactcatcgcagtactgttgtaattcattaagc
attctgccgacatggaagccatcacagacggcatgatgaacctgaatcgccagcggcatcagcaccttgtcgccttgcgtataatatttgcccatagtgaaaacgggggcga
agaagttgtccatattggccacgtttaaatcaaaactggtgaaactcacccagggattggctgagacgaaaaacatattctcaataaaccctttagggaaataggccaggtttt
caccgtaacacgccacatcttgcgaatatatgtgtagaaactgccggaaatcgtcgtggtattcactccagagcgatgaaaacgtttcagtttgctcatggaaaacggtgtaac
aagggtgaacactatcccatatcaccagctcaccgtctttcattgccatacggaactccggatgagcattcatcaggcgggcaagaatgtgaataaaggccggataaaactt
gtgcttatttttctttacggtctttaaaaaggccgtaatatccagctgaacggtctggttataggtacattgagcaactgactgaaatgcctcaaaatgttctttacgatgccattgg
gatatatcaacggtggtatatccagtgatttttttctccattttagcttccttagctcctgaaaatctcgataactcaaaaaatacgcccggtagtgatcttatttcattatggtgaaagt
tggaacctcttacgtgccgatcaacgtctcattttcgccagatatcgacgtctaagaaaccattattatcatgacattaacctataaaaataggcgtatcacgaggccctttcgtct
tcac

Appendix Figure S10 | pUClacZ1 sequence and map

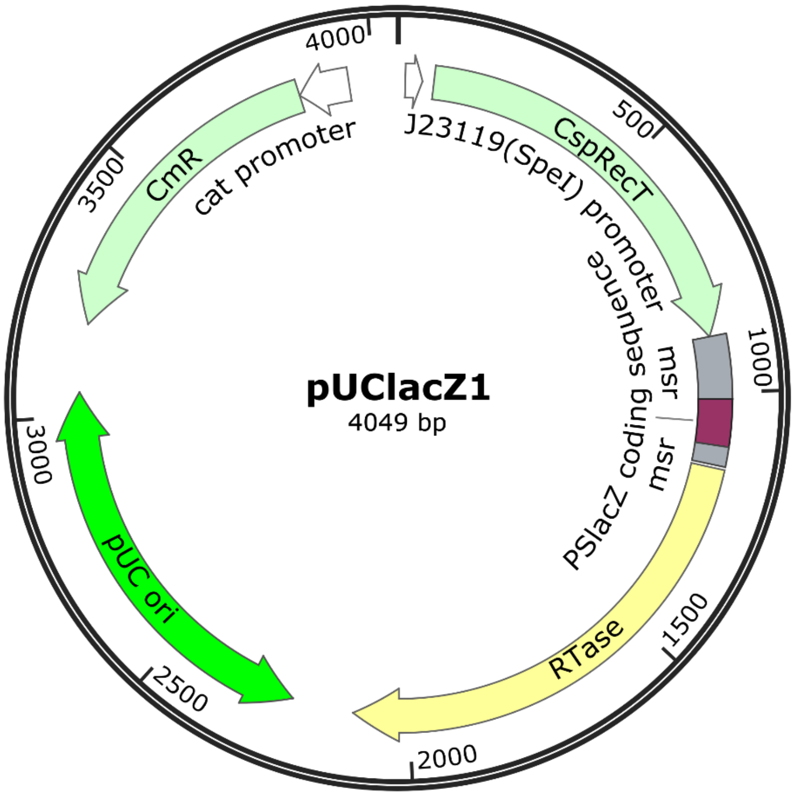

ctcgagatgctagcaattgacagctagctcagtcctaggtataatactagtattgattcagaggtataaaacgaatgaaccaaatcgtgaagttcactgacgactctggcctgg
cggttcaagttactccagacgatgttcgccgttatatctgtgagaacgctactgaaaaagaggtgggcctctttctgcaactctgtcagactcaacgtctgaatccgtttgtgaaa
gacgcttacctggtgaaatacggcggtgctccagcttctatgattacttcctatcaagtttttaaccgtcgcgcgtgtcgtgatgctaactatgatggtatcaaatctggtgtggtt
gttctgcgtgacggtgatgttgtgcataaacgtggtgctgcgtgctacaaaaaggcgggtgaggagctcatcggtggttgggcggaagttcgctttaaggatggccgcgag
actgcgtatgctgaggtggcgctcgacgactattccaccggcaaatctaattgggcgaaaatgccgggtgttatgatcgaaaaatgcgcgaaggctgctgcttggcgcctcg
cgttcccggacacttttcagggcatgtacgctgcggaggaaatggatcaagcgcaacagccagaacaggtgcgcgctcaggcggagcaaccagtggatctccagccaat
ccgcgaactcttcaagccatattgcgaacacttcggcatcactccggctgagggtatgactgctgtttgtggtgcggtgggcgctgaaggcatgcactctatgaccgagcag
caagctcgccgtgctcgcgcttggatggaggaagaaatggctgcgccagctgtggaagcggagtatgaggttgttgacgagggcgaggtgttttaatcagcaggacgcac
tgaccttaattaaatgcgcacccttagcgagaggtttatcattaaggtcaacctctggatgttgtttcggcatcctgcattgaatctgagttactgtctgttttcctgaattcggggat
gtgctgcaaggcgattaagttgggtaacgccagggttttATcagtcacgacgttgtaaaacgacggccagtgaatccgtaatcatggaattcaggaaacccgtttttctgac
gtaagggtgcgcaactttcatgaaatccgctgaatatttgaacacttttagattgagaaatctcggcctacctgtcatgaacaatttgcatgacatgtctaaggcgactcgcatat
ctgttgaaacacttcggttgttaatctatacagctgattttcgctataggatctacactgtagaaaagaaaggcccagagaagagaatgagaaccatttaccaaccttctcgaga
acttaaagccttacaaggatgggttctacgtaacattttagataaactgtcgtcatctcctttttctattggatttgaaaagcaccaatctattttgaataatgctaccccgcatattgg
ggcaaactttatactgaatattgatttggaggatttttcccaagtttaactgctaacaaagtttttggagtgttccattctcttggttataatcgactaatatcttcagttttgacaaaaa
tatgttgttataaaaatctgctaccacaaggtgctccatcatcacctaaattagctaatctaatatgttctaaacttgattatcgtattcagggttatgcaggtagtcggggcttgatat
atacgagatatgccgatgacctcaccttatctgcacagtctatgaaaaaggttgttaaagcacgtgatttttttattttctataatcccaagtgaaggattggttattaactcaaaaaa
aacttgtattagtgggcctcgtagtcagaggaaagttacaggtttagttatttcacaagagaaagttgggataggtagagaaaaatataaagaaattagagcaaagatacatca
tatattttgcggtaagtcttctgagatagaacacgttaggggatggttgtcatttattttaagtgtggattcaaaaagccataggagattaataacttatattagcaaattagaaaaa
aaatatggaaagaaccctttaaataaagcgaagacctaaggatccggttgatAACGCGTGCTAGAGGCATcaaataaaacgaaaggctcagtcgaaagact
gggcctttcgttttatctgttgtttgtcggtgaacgctctcctgagtaggacaaatccgccgccctagacctagggcgttcggctgcggcgagcggtatcagctcactcaaagg
cggtaatacggttatccacagaatcaggggataacgcaggaaagaacatgtgagcaaaaggccagcaaaaggccaggaaccgtaaaaaggccgcgttgctggcgttttt
ccataggctccgcccccctgacgagcatcacaaaaatcgacgctcaagtcagaggtggcgaaacccgacaggactataaagataccaggcgtttccccctggaagctccc

tcgtgcgctctcctgttccgaccctgccgcttaccggatacctgtccgcctttctcccttcgggaagcgtggcgctttctcaTAgctcacgctgtaggtatctcagttcggtgta
ggtcgttcgctccaagctgggctgtgtgcacgaaccccccgttcagcccgaccgctgcgccttatccggtaactatcgtcttgagtccaacccggtaagacacgacttatcg
ccactggcagcagccactggtaacaggattagcagagcgaggtatgtaggcggtgctacagagttcttgaagtggtggcctaactacggctacactagaagAacagtattt
ggtatctgcgctctgctgaagccagttaccttcggaaaaagagttggtagctcttgatccggcaaacaaaccaccgctggtagcggtggtttttttgtttgcaagcagcagatta
cgcgcagaaaaaaaggatctcaagaagatcctttgatcttttctacggggtctgacgctcagtggaacgaaaactcacgttaagggattttggtcatgactagtgcttggattct
caccaataaaaaacgcccggcggcaaccgagcgttctgaacaaatccagatggagttctgaggtcattactggatctatcaacaggagtccaagcgagctcgatatcaaatt
acgccccgccctgccactcatcgcagtactgttgtaattcattaagcattctgccgacatggaagccatcacagacggcatgatgaacctgaatcgccagcggcatcagcac
cttgtcgccttgcgtataatatttgcccatggtgaaaacgggggcgaagaagttgtccatattggccacgtttaaatcaaaactggtgaaactcacccagggattggctgagac
gaaaaacatattctcaataaaccctttagggaaataggccaggttttcaccgtaacacgccacatcttgcgaatatatgtgtagaaactgccggaaatcgtcgtggtattcactc
cagagcgatgaaaacgtttcagtttgctcatggaaaacggtgtaacaagggtgaacactatcccatatcaccagctcaccgtctttcattgccatacggaattccggatgagca
ttcatcaggcgggcaagaatgtgaataaaggccggataaaacttgtgcttatttttctttacggtctttaaaaaggccgtaatatccagctgaacggtctggttataggtacattga
gcaactgactgaaatgcctcaaaatgttctttacgatgccattgggatatatcaacggtggtatatccagtgatttttttctccattttagcttccttagctcctgaaaatctcgataac
tcaaaaaatacgcccggtagtgatcttatttcattatggtgaaagttggaacctcttacgtgccgatcaacgtctcattttcgccagatatcgacgtctaagaaaccattattatcat
gacattaacctataaaaataggcgtatcacgaggccctttcgtcttcac

Appendix Figure S11 | pUClacZ2 sequence and map

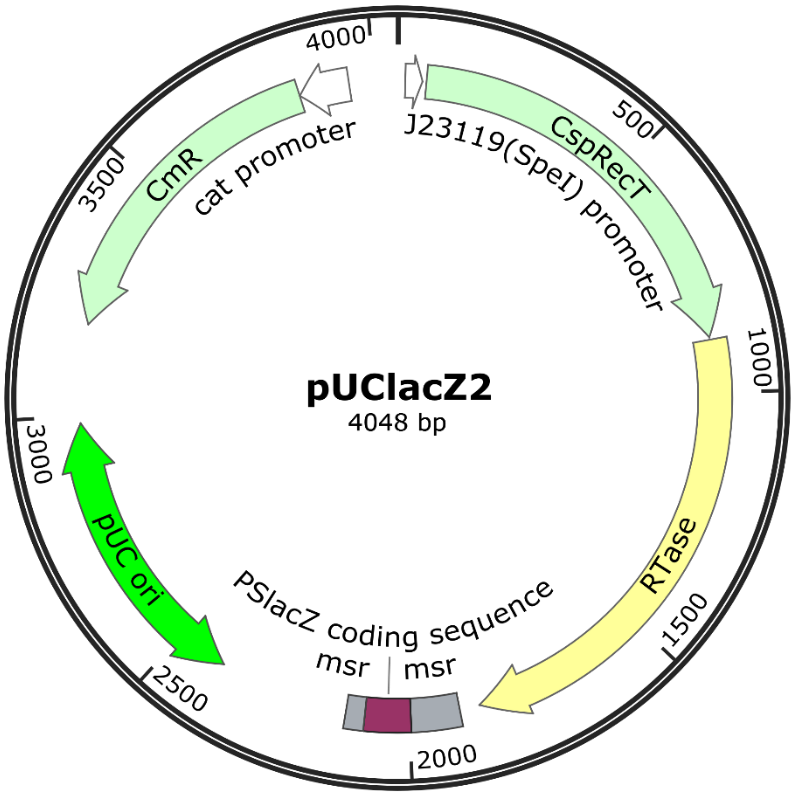

ctcgagatgctagcaattgacagctagctcagtcctaggtataatactagtattgattcagaggtataaaacgaatgaaccaaatcgtgaagttcactgacgactctggcctgg
cggttcaagttactccagacgatgttcgccgttatatctgtgagaacgctactgaaaaagaggtgggcctctttctgcaactctgtcagactcaacgtctgaatccgtttgtgaaa
gacgcttacctggtgaaatacggcggtgctccagcttctatgattacttcctatcaagtttttaaccgtcgcgcgtgtcgtgatgctaactatgatggtatcaaatctggtgtggtt
gttctgcgtgacggtgatgttgtgcataaacgtggtgctgcgtgctacaaaaaggcgggtgaggagctcatcggtggttgggcggaagttcgctttaaggatggccgcgag
actgcgtatgctgaggtggcgctcgacgactattccaccggcaaatctaattgggcgaaaatgccgggtgttatgatcgaaaaatgcgcgaaggctgctgcttggcgcctcg
cgttcccggacacttttcagggcatgtacgctgcggaggaaatggatcaagcgcaacagccagaacaggtgcgcgctcaggcggagcaaccagtggatctccagccaat
ccgcgaactcttcaagccatattgcgaacacttcggcatcactccggctgagggtatgactgctgtttgtggtgcggtgggcgctgaaggcatgcactctatgaccgagcag
caagctcgccgtgctcgcgcttggatggaggaagaaatggctgcgccagctgtggaagcggagtatgaggttgttgacgagggcgaggtgttttaaactttcatgaaatcc
gctgaatatttgaacacttttagattgagaaatctcggcctacctgtcatgaacaatttgcatgacatgtctaaggcgactcgcatatctgttgaaacacttcggttgttaatctata
cagctgattttcgctataggatctacactgtagaaaagaaaggcccagagaagagaatgagaaccatttaccaaccttctcgagaacttaaagccttacaaggatgggttcta
cgtaacattttagataaactgtcgtcatctcctttttctattggatttgaaaagcaccaatctattttgaataatgctaccccgcatattggggcaaactttatactgaatattgatttgg
aggatttttttcccaagtttaactgctaacaaagtttttggagtgttccattctcttggttataatcgactaatatcttcagttttgacaaaaatatgttgttataaaaatctgctaccacaa
ggtgctccatcatcacctaaattagctaatctaatatgttctaaacttgattatcgtattcagggttatgcaggtagtcggggcttgatatatacgagatatgccgatgacctcacct
tatctgcacagtctatgaaaaaggttgttaaagcacgtgatttttttattttctataatcccaagtgaaggattggttattaactcaaaaaaaaacttgtattagtgggcctcgtagtcag
aggaaagttacaggtttagttatttcacaagagaaagttgggataggtagagaaaaatataaagaaattagagcaaagatacatcatatattttgcggtaagtcttctgagatag
aacacgttaggggatggttgtcatttattttaagtgtggattcaaaaagccataggagattaataacttatattagcaaattagaaaaaaaatatggaaagaacccttttaaataaag
cgaagacctaaggatccggttgatatcagcaggacgcactgaccttaattaaatgcgcacccttagcgagaggtttatcattaaggtcaacctctggatgttgtttcggcatcct
gcattgaatctgagttactgtctgttttcctgaattcggggatgtgctgcaaggcgattaagttgggtaacgccagggttttATcagtcacgacgttgtaaaacgacggccagt
gaatccgtaatcatggaattcaggaaacccgtttttttctgacgtaagggtgcgcaacgcgtgctagaggcatcaaataaaacgaaaggctcagtcgaaagactgggcctttc
gttttatctgttgtttgtcggtgaacgctctcctgagtaggacaaatccgccgccctagacctaggcgttcggctgcggcgagcggtatcagctcactcaaaggcggtaatac
ggttatccacagaatcaggggataacgcaggaaagaacatgtgagcaaaaggccagcaaaaggccaggaaccgtaaaaaggccgcgttgctggcgtttttccataggct
ccgcccccctgacgagcatcacaaaaatcgacgctcaagtcagaggtggcgaaacccgacaggactataaagataccaggcgtttccccctggaagctccctcgtgcgct
ctcctgttccgaccctgccgcttaccggatacctgtccgcctttctcccttcgggaagcgtggcgctttctcatagctcacgctgtaggtatctcagttcggtgtaggtcgttcgc

tccaagctgggctgtgtgcacgaaccccccgttcagcccgaccgctgcgccttatccggtaactatcgtcttgagtccaacccggtaagacacgacttatcgccactggcag
cagccactggtaacaggattagcagagcgaggtatgtaggcggtgctacagagttcttgaagtggtggcctaactacggctacactagaagAacagtatttggtatctgcg
ctctgctgaagccagttaccttcggaaaaagagttggtagctcttgatccggcaaacaaaccaccgctggtagcggtggtttttttgtttgcaagcagcagattacgcgcagaa
aaaaaggatctcaagaagatcctttgatcttttctacggggtctgacgctcagtggaacgaaaactcacgttaagggattttggtcatgactagtgcttggattctcaccaataaa
aaacgcccggcggcaaccgagcgttctgaacaaatccagatggagttctgaggtcattactggatctatcaacaggagtccaagcgagctcgatatcaaattacgccccgc
cctgccactcatcgcagtactgttgtaattcattaagcattctgccgacatggaagccatcacagacggcatgatgaacctgaatcgccagcggcatcagcaccttgtcgcctt
gcgtataatatttgcccatggtgaaaacgggggcgaagaagttgtccatattggccacgtttaaatcaaaactggtgaaactcacccagggattggctgagacgaaaaacat
attctcaataaaccctttagggaaataggccaggttttcaccgtaacacgccacatcttgcgaatatatgtgtagaaactgccggaaatcgtcgtggtattcactccagagcgat
gaaaacgtttcagtttgctcatggaaaacggtgtaacaagggtgaacactatcccatatcaccagctcaccgtctttcattgccatacggaattccggatgagcattcatcagg
cgggcaagaatgtgaataaaggccggataaaacttgtgcttatttttctttacggtctttaaaaaggccgtaatatccagctgaacggtctggttataggtacattgagcaactga
ctgaaatgcctcaaaatgttctttacgatgccattgggatatatcaacggtggtatatccagtgatttttttctccattttagcttccttagctcctgaaaatctcgataactcaaaaaa
tacgcccggtagtgatcttatttcattatggtgaaagttggaacctcttacgtgccgatcaacgtctcattttcgccagatatcgacgtctaagaaaccattattatcatgacattaa
cctataaaaataggcgtatcacgaggccctttcgtcttcac

Appendix Figure S12 | pUClacZ3 sequence and map

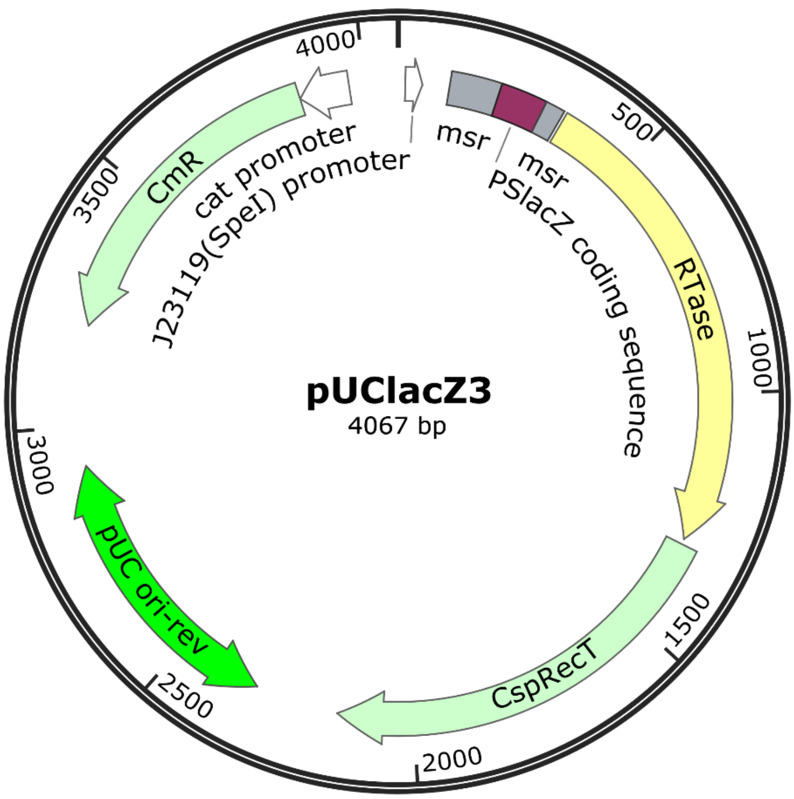

ctcgagatgctagcaattgacagctagctcagtcctaggtataatactagtatcagcaggacgcactgaccttaattaatcagcaggacgcactgaccttaattaaatgcgcac
ccttagcgagaggtttatcattaaggtcaacctctggatgttgtttcggcatcctgcattgaatctgagttactgtctgttttcctgaattcggggatgtgctgcaaggcgattaagt
tgggtaacgccagggttttATcagtcacgacgttgtaaaacgacggccagtgaatccgtaatcatggaattcaggaaacccgtttttctgacgtaagggtgcgcaactttcat
gaaatccgctgaatatttgaacacttttagattgagaaatctcggcctacctgtcatgaacaatttgcatgacatgtctaaggcgactcgcatatctgttgaaacacttcggttgtta
atctatacagctgattttcgctataggatctacactgtagaaaagaaaggcccagagaagagaatgagaaccatttaccaaccttctcgagaacttaaagccttacaaggatg
ggttctacgtaacattttagataaactgtcgtcatctcctttttctattggatttgaaaagcaccaatctattttgaataatgctaccccgcatattggggcaaactttatactgaatatt
gatttggaggatttttcccaagtttaactgctaacaaagtttttggagtgttccattctcttggttataatcgactaatatcttcagttttgacaaaaatatgttgttataaaaatctgcta
ccacaaggtgctccatcatcacctaaattagctaatctaatatgttctaaacttgattatcgtattcagggttatgcaggtagtcggggcttgatatatacgagatatgccgatgac
ctcaccttatctgcacagtctatgaaaaaggttgttaaagcacgtgatttttattttctataatcccaagtgaaggattggttattaactcaaaaaaaacttgtattagtgggcctcgt
agtcagaggaaagttacaggtttagttatttcacaagagaaagttgggataggtagagaaaaatataaagaaattagagcaaagatacatcatatattttgcggtaagtcttctg
agatagaacacgttaggggatggttgtcatttattttaagtgtggattcaaaaagccataggagattaataacttatattagcaaattagaaaaaaaatatggaaagaaccctttta
aataaagcgaagacctaaggatccggttgatattgattcagaggtataaaacgaatgaaccaaatcgtgaagttcactgacgactctggcctggcggttcaagttactccaga
cgatgttcgccgttatatctgtgagaacgctactgaaaaagaggtgggcctctttctgcaactctgtcagactcaacgtctgaatccgtttgtgaaagacgcttacctggtgaaa
tacggcggtgctccagcttctatgattacttcctatcaagtttttaaccgtcgcgcgtgtcgtgatgctaactatgatggtatcaaatctggtgtggttgttctgcgtgacggtgatg
ttgtgcataaacgtggtgctgcgtgctacaaaaaggcgggtgaggagctcatcggtggttgggcggaagttcgctttaaggatggccgcgagactgcgtatgctgaggtgg
cgctcgacgactattccaccggcaaatctaattgggcgaaaatgccgggtgttatgatcgaaaaatgcgcgaaggctgctgcttggcgcctcgcgttcccggacacttttca
gggcatgtacgctgcggaggaaatggatcaagcgcaacagccagaacaggtgcgcgctcaggcggagcaaccagtggatctccagccaatccgcgaactcttcaagcc
atattgcgaacacttcggcatcactccggctgagggtatgactgctgtttgtggtgcggtgggcgctgaaggcatgcactctatgaccgagcagcaagctcgccgtgctcgc
gcttggatggaggaagaaatggctgcgccagctgtggaagcggagtatgaggttgttgacgagggcgaggtgttttaaacgcgtgctagaggcatcaaataaaacgaaag
gctcagtcgaaagactgggcctttcgttttatctgttgtttgtcggtgaacgctctcctgagtaggacaaatccgccgcccttaacgtgagttttcgttccactgagcgtcagacc
ccgtagaaaagatcaaaggatcttcttgagatcctttttttctgcgcgtaatctgctgcttgcaaacaaagaaaccaccgctaccagcggtggtttgtttgccggatcaagagct
accaactctttttccgaaggtaactggcttcagcagagcgcagataccaaatactgtTcttctagtgtagccgtagttaggccaccacttcaagaactctgtagcaccgcctac
atacctcgctccgctaatcctgttaccagtggctgctgccagtggcgataagtcgtgtcttaccgggttggactcaagacgatagttaccggataaggcgcagcggtcgggc
tgaacggggggttcgtgcacacagcccagcttggagcgaacgacctacaccgaactgagatacctacagcgtgagcTAtgagaaagcgccacgcttcccgaagggag

aaaggcggacaggtatccggtaagcggcagggtcggaacaggagagcgcacgagggagcttccagggggaaacgcctggtatctttatagtcctgtcgggtttcgccac
ctctgacttgagcgtcgatttttgtgatgctcgtcaggggggcggagcctatggaaaaacgccagcaacgcggcctctttacggttcctggccttttgctggccttttgctcaca
tgttctttcctgcgttatcccctgattctgtggataaccgtattaccgcctttgagtgagctgataccgctcgccgcagccgaacgcctaggtctaggtcatgactagtgcttgga
ttctcaccaataaaaaacgcccggcggcaaccgagcgttctgaacaaatccagatggagttctgaggtcattactggatctatcaacaggagtccaagcgagctcgatatca
aattacgccccgccctgccactcatcgcagtactgttgtaattcattaagcattctgccgacatggaagccatcacagacggcatgatgaacctgaatcgccagcggcatcag
caccttgtcgccttgcgtataatatttgcccatggtgaaaacgggggcgaagaagttgtccatattggccacgtttaaatcaaaactggtgaaactcacccagggattggctga
gacgaaaaacatattctcaataaaccctttagggaaataggccaggttttcaccgtaacacgccacatcttgcgaatatatgtgtagaaactgccggaaatcgtcgtggtattca
ctccagagcgatgaaaacgtttcagtttgctcatggaaaacggtgtaacaagggtgaacactatcccatatcaccagctcaccgtctttcattgccatacggaattccggatga
gcattcatcaggcgggcaagaatgtgaataaaggccggataaaacttgtgcttatttttctttacggtctttaaaaaggccgtaatatccagctgaacggtctggttataggtaca
ttgagcaactgactgaaatgcctcaaaatgttctttacgatgccattgggatatatcaacggtggtatatccagtgatttttttctccattttagcttccttagctcctgaaaatctcga
taactcaaaaaatacgcccggtagtgatcttatttcattatggtgaaagttggaacctcttacgtgccgatcaacgtctcattttcgccagatatcgacgtctaagaaaccattatta
tcatgacattaacctataaaaataggcgtatcacgaggccctttcgtcttcac

Appendix Figure S13 | pUCung sequence and map

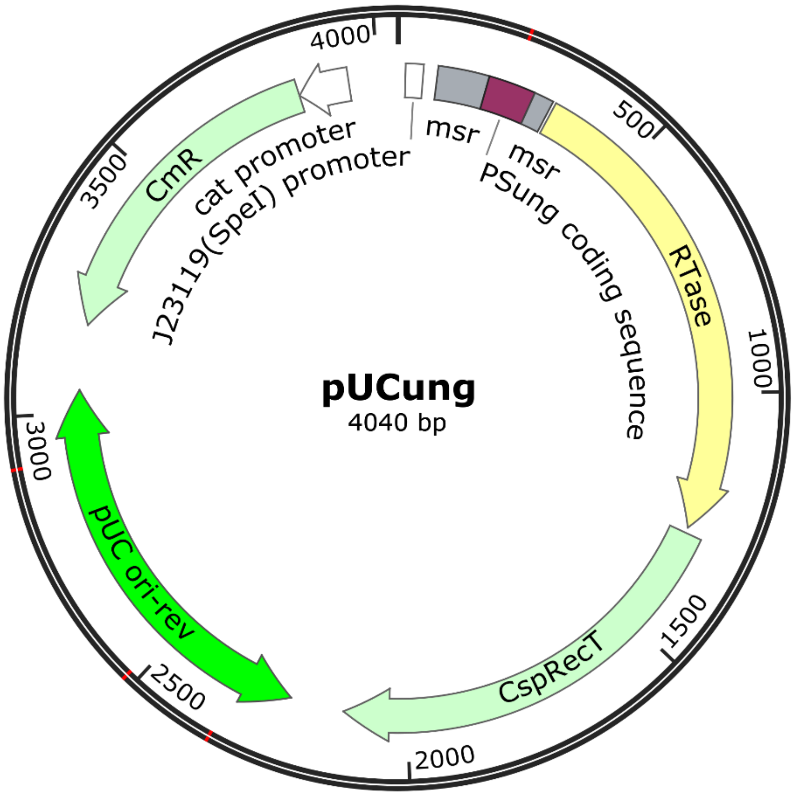

ctcgagatgctagcaattgacagctagctcagtcctaggtataatactagtatcagcaggacgcactgaccttaattaaatgcgcacccttagcgagaggtttatcattaaggtc
aacctctggatgttgtttcggcatcctgcattgaatctgagttactgtctgttttcctgaattcccatgattagggcgggtgaagcccggaatagtattttccagctcttactacatat
tcaataatgacggaggaatggcaatgccgggacgaacgaattcaggaaacccgtttttctgacgtaagggtgcgcaactttcatgaaatccgctgaatatttgaacactttta
gattgagaaatctcggcctacctgtcatgaacaatttgcatgacatgtctaaggcgactcgcatatctgttgaaacacttcggttgttaatctatacagctgattttcgctataggat
ctacactgtagaaaagaaaggcccagagaagagaatgagaaccatttaccaaccttctcgagaacttaaagccttacaaggatgggttctacgtaacattttagataaactgtc
gtcatctccttttctattggatttgaaaagcaccaatctattttgaataatgctaccccgcatattggggcaaactttatactgaatattgatttggaggatttttcccaagtttaactg
ctaacaaagtttttggagtgttccattctcttggttataatcgactaatatcttcagttttgacaaaaatatgttgttataaaaatctgctaccacaaggtgctccatcatcacctaaatt
agctaatctaatatgttctaaacttgattatcgtattcagggttatgcaggtagtcggggcttgatatatacgagatatgccgatgacctcaccttatctgcacagtctatgaaaaa
ggttgttaaagcacgtgatttttattttctataatcccaagtgaaggattggttattaactcaaaaaaaacttgtattagtgggcctcgtagtcagaggaaagttacaggtttagtta
tttcacaagagaaagttgggataggtagagaaaaatataaagaaattagagcaaagatacatcatatattttgcggtaagtcttctgagatagaacacgttaggggatggttgtc
atttattttaagtgtggattcaaaaagccataggagattaataacttatattagcaaattagaaaaaaaatatggaaagaaccctttaaataaagcgaagacctaaggatccggtt
gatattgattcagaggtataaaacgaatgaaccaaatcgtgaagttcactgacgactctggcctggcggttcaagttactccagacgatgttcgccgttatatctgtgagaacg
ctactgaaaaagaggtgggcctctttctgcaactctgtcagactcaacgtctgaatccgtttgtgaaagacgcttacctggtgaaatacggcggtgctccagcttctatgattact
tcctatcaagtttttaaccgtcgcgcgtgtcgtgatgctaactatgatggtatcaaatctggtgtggttgttctgcgtgacggtgatgttgtgcataaacgtggtgctgcgtgctac
aaaaaggcgggtgaggagctcatcggtggttgggcggaagttcgctttaaggatggccgcgagactgcgtatgctgaggtggcgctcgacgactattccaccggcaaatc
taattgggcgaaaatgccgggtgttatgatcgaaaaatgcgcgaaggctgctgcttggcgcctcgcgttcccggacacttttcagggcatgtacgctgcggaggaaatggat
caagcgcaacagccagaacaggtgcgcgctcaggcggagcaaccagtggatctccagccaatccgcgaactcttcaagccatattgcgaacacttcggcatcactccgg
ctgagggtatgactgctgtttgtggtgcggtgggcgctgaaggcatgcactctatgaccgagcagcaagctcgccgtgctcgcgcttggatggaggaagaaatggctgcg
ccagctgtggaagcggagtatgaggttgttgacgagggcgaggtgttttaaacgcgtgctagaggcatcaaataaaacgaaaggctcagtcgaaagactgggcctttcgttt
tatctgttgtttgtcggtgaacgctctcctgagtaggacaaatccgccgcccttaacgtgagttttcgttccactgagcgtcagaccccgtagaaaagatcaaaggatcttcttga
gatcctttttttctgcgcgtaatctgctgcttgcaaacaaagaaaccaccgctaccagcggtggtttgtttgccggatcaagagctaccaactctttttccgaaggtaactggcttc
agcagagcgcagataccaaatactgtTcttctagtgtagccgtagttaggccaccacttcaagaactctgtagcaccgcctacatacctcgctccgctaatcctgttaccagtg
gctgctgccagtggcgataagtcgtgtcttaccgggttggactcaagacgatagttaccggataaggcgcagcggtcgggctgaacggggggttcgtgcacacagccca
gcttggagcgaacgacctacaccgaactgagatacctacagcgtgagcTAtgagaaagcgccacgcttcccgaagggagaaaggcggacaggtatccggtaagcgg

cagggtcggaacaggagagcgcacgagggagcttccagggggaaacgcctggtatctttatagtcctgtcgggtttcgccacctctgacttgagcgtcgatttttgtgatgct
cgtcaggggggcggagcctatggaaaaacgccagcaacgcggcctctttacggttcctggccttttgctggccttttgctcacatgttctttcctgcgttatcccctgattctgtg
gataaccgtattaccgcctttgagtgagctgataccgctcgccgcagccgaacgcctaggtctaggtcatgactagtgcttggattctcaccaataaaaaacgcccggcggc
aaccgagcgttctgaacaaatccagatggagttctgaggtcattactggatctatcaacaggagtccaagcgagctcgatatcaaattacgccccgccctgccactcatcgca
gtactgttgtaattcattaagcattctgccgacatggaagccatcacagacggcatgatgaacctgaatcgccagcggcatcagcaccttgtcgccttgcgtataatatttgccc
atggtgaaaacgggggcgaagaagttgtccatattggccacgtttaaatcaaaactggtgaaactcacccagggattggctgagacgaaaaacatattctcaataaaccctttt
agggaaataggccaggttttcaccgtaacacgccacatcttgcgaatatatgtgtagaaactgccggaaatcgtcgtggtattcactccagagcgatgaaaacgtttcagtttg
ctcatggaaaacggtgtaacaagggtgaacactatcccatatcaccagctcaccgtctttcattgccatacggaattccggatgagcattcatcaggcgggcaagaatgtgaa
taaaggccggataaaacttgtgcttatttttctttacggtctttaaaaaggccgtaatatccagctgaacggtctggttataggtacattgagcaactgactgaaatgcctcaaaat
gttctttacgatgccattgggatatatcaacggtggtatatccagtgatttttttctccattttagcttccttagctcctgaaaatctcgataactcaaaaaatacgcccggtagtgatc
ttatttcattatggtgaaagttggaacctcttacgtgccgatcaacgtctcattttcgccagatatcgacgtctaagaaaccattattatcatgacattaacctataaaaataggcgt
atcacgaggccctttcgtcttcac

Appendix Figure S14 | pUCbetI sequence and map

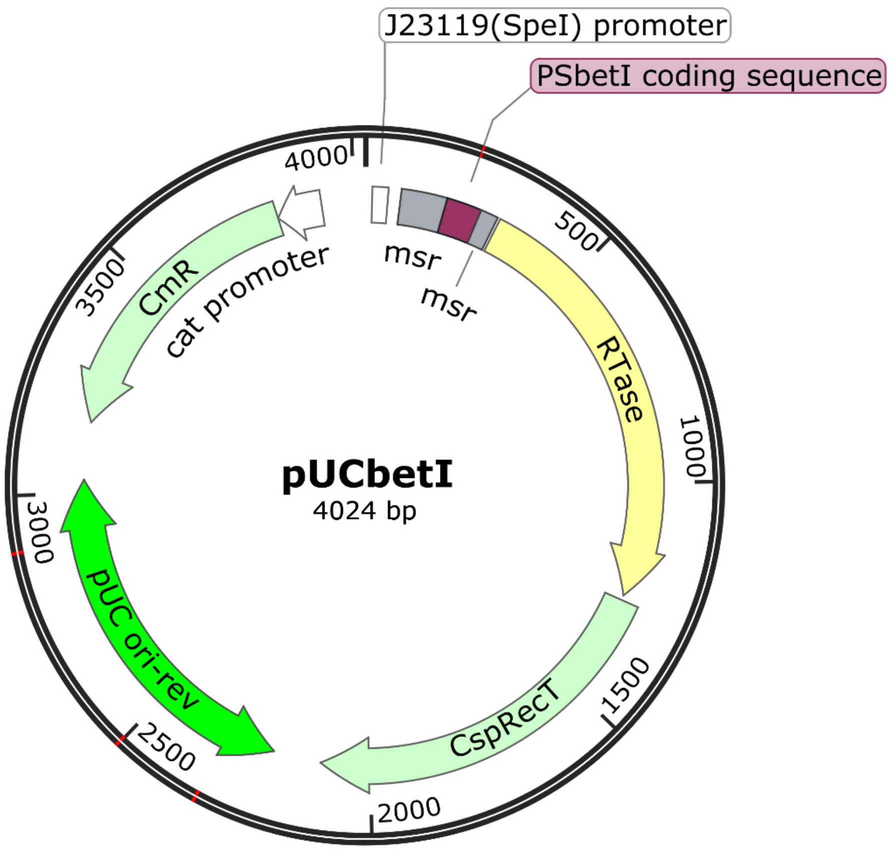

ctcgagatgctagcaattgacagctagctcagtcctaggtataatactagtatcagcaggacgcactgaccttaattaaatgcgcacccttagcgagaggtttatcattaaggtc
aacctctggatgttgtttcggcatcctgcattgaatctgagttactgtctgttttcctgaattcagttgtctgcgccggatcgactgcatccccaatttgggcttttcgccactccattc
atcagcggtgtttatctattgaattcaggaaacccgtttttctgacgtaagggtgcgcaactttcatgaaatccgctgaatatttgaacacttttagattgagaaatctcggcctac
ctgtcatgaacaatttgcatgacatgtctaaggcgactcgcatatctgttgaaacacttcggttgttaatctatacagctgattttcgctataggatctacactgtagaaaagaaag
gcccagagaagagaatgagaaccatttaccaaccttctcgagaacttaaagccttacaaggatgggttctacgtaacattttagataaactgtcgtcatctccttttctattggat
ttgaaaagcaccaatctattttgaataatgctaccccgcatattggggcaaactttatactgaatattgatttggaggatttttcccaagtttaactgctaacaaagtttttggagtgt
tccattctcttggttataatcgactaatatcttcagttttgacaaaaatatgttgttataaaaatctgctaccacaaggtgctccatcatcacctaaattagctaatctaatatgttctaa
acttgattatcgtattcagggttatgcaggtagtcggggcttgatatatacgagatatgccgatgacctcaccttatctgcacagtctatgaaaaaggttgttaaagcacgtgattt
tttattttctataatcccaagtgaaggattggttattaactcaaaaaaaacttgtattagtgggcctcgtagtcagaggaaagttacaggtttagttatttcacaagagaaagttggg
ataggtagagaaaaatataaagaaattagagcaaagatacatcatatattttgcggtaagtcttctgagatagaacacgttaggggatggttgtcatttattttaagtgtggattca
aaaagccataggagattaataacttatattagcaaattagaaaaaaaatatggaaagaaccctttaaataaagcgaagacctaaggatccggttgatattgattcagaggtata
aaacgaatgaaccaaatcgtgaagttcactgacgactctggcctggcggttcaagttactccagacgatgttcgccgttatatctgtgagaacgctactgaaaaagaggtggg
cctctttctgcaactctgtcagactcaacgtctgaatccgtttgtgaaagacgcttacctggtgaaatacggcggtgctccagcttctatgattacttcctatcaagtttttaaccgt
cgcgcgtgtcgtgatgctaactatgatggtatcaaatctggtgtggttgttctgcgtgacggtgatgttgtgcataaacgtggtgctgcgtgctacaaaaaggcgggtgagga
gctcatcggtggttgggcggaagttcgctttaaggatggccgcgagactgcgtatgctgaggtggcgctcgacgactattccaccggcaaatctaattgggcgaaaatgcc
gggtgttatgatcgaaaaatgcgcgaaggctgctgcttggcgcctcgcgttcccggacacttttcagggcatgtacgctgcggaggaaatggatcaagcgcaacagccag
aacaggtgcgcgctcaggcggagcaaccagtggatctccagccaatccgcgaactcttcaagccatattgcgaacacttcggcatcactccggctgagggtatgactgctg
tttgtggtgcggtgggcgctgaaggcatgcactctatgaccgagcagcaagctcgccgtgctcgcgcttggatggaggaagaaatggctgcgccagctgtggaagcgga
gtatgaggttgttgacgagggcgaggtgttttaaacgcgtgctagaggcatcaaataaaacgaaaggctcagtcgaaagactgggcctttcgttttatctgttgtttgtcggtga
acgctctcctgagtaggacaaatccgccgcccttaacgtgagttttcgttccactgagcgtcagaccccgtagaaaagatcaaaggatcttcttgagatcctttttttctgcgcgt
aatctgctgcttgcaaacaaagaaaccaccgctaccagcggtggtttgtttgccggatcaagagctaccaactctttttccgaaggtaactggcttcagcagagcgcagatac
caaatactgtTcttctagtgtagccgtagttaggccaccacttcaagaactctgtagcaccgcctacatacctcgctccgctaatcctgttaccagtggctgctgccagtggcg
ataagtcgtgtcttaccgggttggactcaagacgatagttaccggataaggcgcagcggtcgggctgaacggggggttcgtgcacacagcccagcttggagcgaacgac

ctacaccgaactgagatacctacagcgtgagcTAtgagaaagcgccacgcttcccgaagggagaaaggcggacaggtatccggtaagcggcagggtcggaacagga gagcgcacgagggagcttccagggggaaacgcctggtatctttatagtcctgtcgggtttcgccacctctgacttgagcgtcgatttttgtgatgctcgtcaggggggcgga gcctatggaaaaacgccagcaacgcggcctctttacggttcctggccttttgctggccttttgctcacatgttctttcctgcgttatcccctgattctgtggataaccgtattaccgc ctttgagtgagctgataccgctcgccgcagccgaacgcctaggtctaggtcatgactagtgcttggattctcaccaataaaaaacgcccggcggcaaccgagcgttctgaac aaatccagatggagttctgaggtcattactggatctatcaacaggagtccaagcgagctcgatatcaaattacgccccgccctgccactcatcgcagtactgttgtaattcatta agcattctgccgacatggaagccatcacagacggcatgatgaacctgaatcgccagcggcatcagcaccttgtcgccttgcgtataatatttgcccatggtgaaaacgggg gcgaagaagttgtccatattggccacgtttaaatcaaaactggtgaaactcacccagggattggctgagacgaaaaacatattctcaataaaccctttagggaaataggccag gttttcaccgtaacacgccacatcttgcgaatatatgtgtagaaactgccggaaatcgtcgtggtattcactccagagcgatgaaaacgtttcagtttgctcatggaaaacggtg taacaagggtgaacactatcccatatcaccagctcaccgtctttcattgccatacggaattccggatgagcattcatcaggcgggcaagaatgtgaataaaggccggataaa acttgtgcttatttttctttacggtctttaaaaaggccgtaatatccagctgaacggtctggttataggtacattgagcaactgactgaaatgcctcaaaatgttctttacgatgccat tgggatatatcaacggtggtatatccagtgatttttttctccattttagcttccttagctcctgaaaatctcgataactcaaaaaatacgcccggtagtgatcttatttcattatggtga aagttggaacctcttacgtgccgatcaacgtctcattttcgccagatatcgacgtctaagaaaccattattatcatgacattaacctataaaaataggcgtatcacgaggccctttc gtcttcac
