# Supplementary material for: Reducing competition between msd and genomic DNA improves retron editing efficiency
Source: EMBO Rep. 2024 Nov 5;25(12):5316–30. doi: 10.1038/s44319-024-00311-6 (PMC11624263; doi:10.1038/s44319-024-00311-6)
Supplement: Supplementary file 7 — Expanded View Figures [file 44319_2024_311_MOESM7_ESM.pdf]

# Expanded View Figures

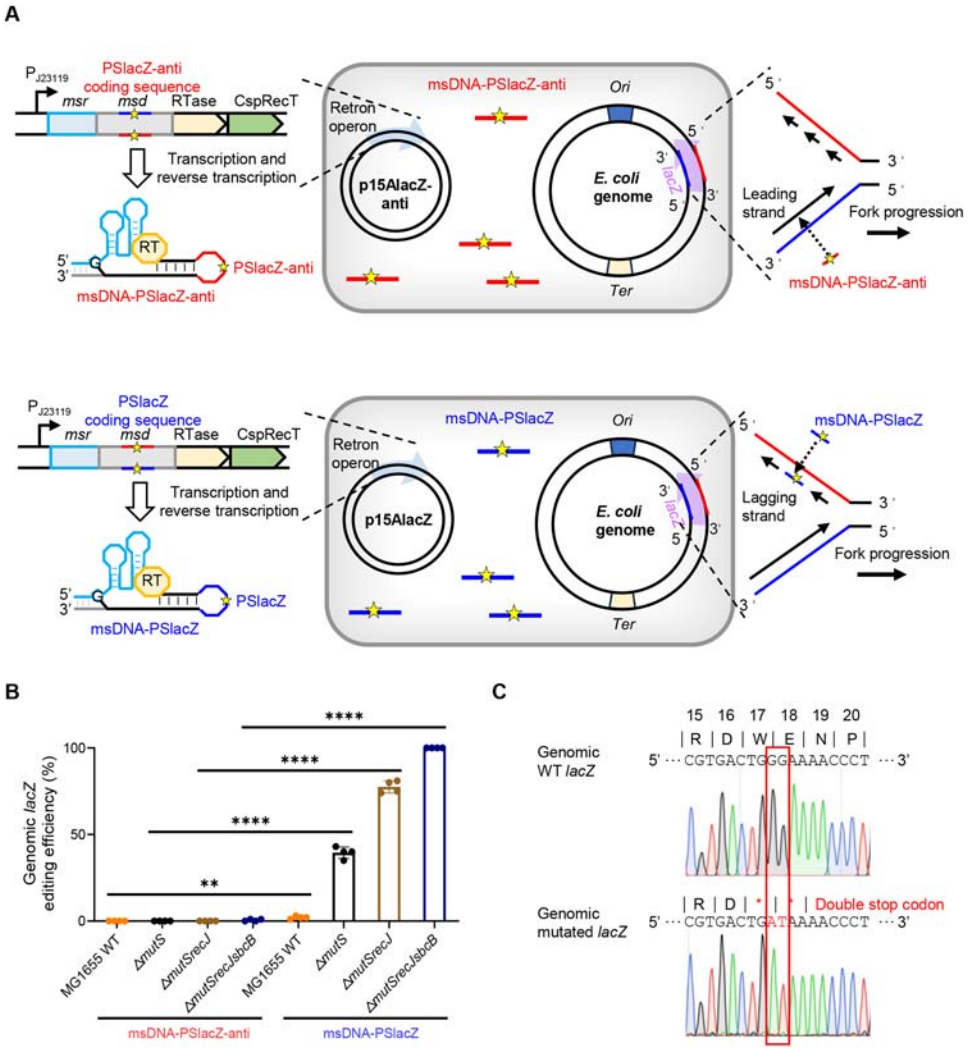


**Figure EV1. Schematic representation of the retron operon and msDNA-mediated genomic *lacZ* editing process.**

(**A**) Top panel: The retron operon is represented by double-stranded DNA labeled as the coding strand and template strand, respectively. A strong constitutive promoter, J23119 (black arrow), drives transcription of the operon, which encodes msDNA (*msr/msd*), reverse transcriptase (*ret*), and CspRecT (*recT*). The coding regions of *msr* and *msd* are depicted as blue and gray rectangles, respectively. A segment within the *msd* coding region (gray rectangle), which shares significant sequence similarity with the genomic *lacZ* gene, is denoted as the PSlacZ-anti coding sequence (PS stands for partial sequence). The blue line aligns with the *lacZ* coding strand, while the red line represents its reverse complement. The mutation sequence is indicated by a yellow star. Transcription and reverse transcription produce msDNA, a hybrid of msr RNA (light blue) and cDNA (black). The msDNA incorporates *lacZ* homologous sequences, designated PSlacZ-anti (red). *E. coli* genomic DNA and plasmid are shown as double-stranded structures. The genomic *ori* and *ter* are marked in blue and yellow, respectively. The *lacZ* gene and its transcription direction are indicated by a purple arrow. During *E. coli* genomic DNA replication, msDNA (red lines) integrates into the single-stranded DNA region at the replication fork through base pairing. Notably, msDNA-PSlacZ-anti has the same sequence as the leading strand. The detailed sequence of the plasmid is provided in Appendix Fig. S1. Bottom panel: The retron operon expresses msDNA-PSlacZ, which incorporates *lacZ* homologous sequences designated PSlacZ (blue). Importantly, PSlacZ is the reverse complement of PSlacZ-anti. PSlacZ shares the same sequence as the lagging strand. (**B**) Effect of strain and editing template on genomic *lacZ* gene editing efficiency at 24 h of incubation. We compared the editing efficiency in WT (no mutation control), Δ*mutS*, Δ*mutSrecJ*, and Δ*mutSrecJsbcB* strains expressing msDNA-PSlacZ-anti or msDNA-PSlacZ. The data are presented as a bar graph, with each bar representing the mean, and error bars indicating the standard deviation. Each data point represents an individual biological replicate ($n = 4$). Statistically significant differences were determined using an unpaired Student's *t*-test. The *p* value for the comparison of the editing efficiency between msDNA-PSlacZ-anti and msDNA-PSlacZ was 0.002714 in strain MG1655. In the Δ*mutS*, Δ*mutSrecJ*, and Δ*mutSrecJsbcB* strains, the *p* value for this comparison was <0.000001. **$P < 0.005$, ****$P < 0.0001$. (**C**) Sequencing results of the genomic *lacZ* sequence of the target region. Mutations change the codons for tryptophan and glutamate at positions 17 and 18 of the LacZ protein from TGG and GAA to stop codons (TGA and TAA). Red letters represent mutated bases. Source data are available online for this figure.


© The Author(s)

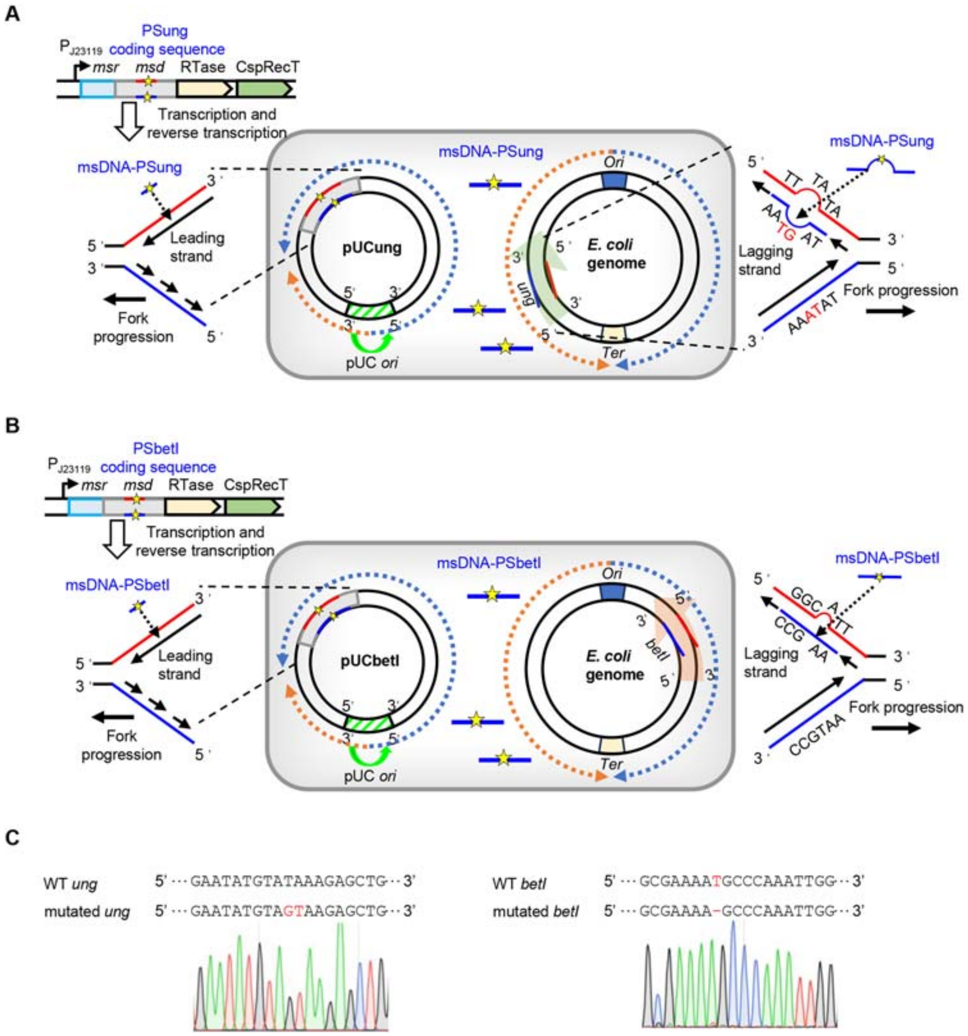


**Figure EV2. Genomic *ung* and *betI* gene editing in Δ*mutSrecJsbcB* strains harboring pUCung or pUCbetI plasmids, respectively.**

(**A**) A 92 bp homologous sequence of the *ung* gene (PS*ung* coding sequence) was inserted into the *msd* region. The msDNA-PSung, aligning with the genomic DNA lagging strand, mediates *ung* gene editing, resulting in double base mutations. Notably, the msDNA-PSung aligns with the pUCung plasmid's leading strand, preventing *msd* editing. The detailed sequence of the plasmid is provided in Appendix Fig. S13. (**B**) A 92 bp homologous sequence of the *betI* gene (PSbetI coding sequence) was inserted into the *msd* region. The msDNA-PSbetI, aligning with the genomic DNA lagging strand, mediates *betI* gene editing, resulting in one base deletion. Notably, the msDNA-PSbetI aligns with the pUCbetI plasmid's leading strand, preventing *msd* editing. The detailed sequence of the plasmid is provided in Appendix Fig. S14. (**C**) Sequencing results of the targeted region in the genomic *ung* and *betI* genes. Four colonies were randomly selected from each plate, and these colonies were pooled separately for each strain (one pool for Δ*mutSrecJsbcB*-pUCung and one pool for Δ*mutSrecJsbcB*-pUCbetI) to increase the number of cells analyzed for mutations. The regions surrounding the targeted sequences in the genomic *ung* and *betI* genes were separately amplified by PCR for each pool, followed by DNA sequencing. The DNA sequencing results reflect the average editing efficiency within each pool. If editing is highly efficient (close to 100%), we expect to see a dominant peak corresponding to the mutated sequence, with a very small or undetectable peak for the wild-type sequence. Conversely, lower editing efficiency will result in a mixture of peaks, representing both mutated and wild-type sequences. Mutated bases are indicated in red.

© The Author(s)
